# Prognostic Value of Procalcitonin in Adult Patients with Sepsis: A Systematic Review and Meta-Analysis

**DOI:** 10.1371/journal.pone.0129450

**Published:** 2015-06-15

**Authors:** Dan Liu, Longxiang Su, Gencheng Han, Peng Yan, Lixin Xie

**Affiliations:** 1 Department of Pulmonary & Critical Care Medicine, Chinese PLA General Hospital, 28 Fuxing Road, Beijing, 100853, China; 2 Medical School, Nankai University, 94 Weijin Road, Tianjin, 300071, China; 3 Department of Respiratory Medicine, Tianjin Medical University General Hospital, Tianjin, 300070, China; 4 Department of Critical Care Medicine, Peking Union Medical College Hospital, Peking Union Medical College & Chinese Academy of Medical Sciences, Beijing, 100005, China; 5 Laboratory of Immunology, Institute of Basic Medical Sciences, Beijing, 100850, China; University of Leicester, UNITED KINGDOM

## Abstract

Procalcitonin (PCT) has been widely investigated for its prognostic value in septic patients. However, studies have produced conflicting results. The purpose of the present meta-analysis is to explore the diagnostic accuracy of a single PCT concentration and PCT non-clearance in predicting all-cause sepsis mortality. We searched PubMed, Embase, Web of Knowledge and the Cochrane Library. Articles written in English were included. A 2 × 2 contingency table was constructed based on all-cause mortality and PCT level or PCT non-clearance in septic patients. Two authors independently evaluated study eligibility and extracted data. The diagnostic value of PCT in predicting prognosis was determined using a bivariate meta-analysis model. We used the Q-test and *I* ^2^ index to test heterogeneity. Twenty-three studies with 3,994 patients were included. An elevated PCT level was associated with a higher risk of death. The pooled relative risk (RR) was 2.60 (95% confidence interval (CI), 2.05–3.30) using a random-effects model (*I* ^2^ = 63.5%). The overall area under the summary receiver operator characteristic (SROC) curve was 0.77 (95% CI, 0.73–0.80), with a sensitivity and specificity of 0.76 (95% CI, 0.67–0.82) and 0.64 (95% CI, 0.52–0.74), respectively. There was significant evidence of heterogeneity for the PCT testing time (*P* = 0.020). Initial PCT values were of limited prognostic value in patients with sepsis. PCT non-clearance was a prognostic factor of death in patients with sepsis. The pooled RR was 3.05 (95% CI, 2.35–3.95) using a fixed-effects model (*I* ^2^ = 37.9%). The overall area under the SROC curve was 0.79 (95% CI, 0.75–0.83), with a sensitivity and specificity of 0.72 (95% CI, 0.58–0.82) and 0.77 (95% CI, 0.55–0.90), respectively. Elevated PCT concentrations and PCT non-clearance are strongly associated with all-cause mortality in septic patients. Further studies are needed to define the optimal cut-off point and the optimal definition of PCT non-clearance for accurate risk assessment.

## Introduction

Sepsis is a life-threatening condition that arises when the body’s response to an infection injures its own tissues and organs [1]. Despite advances in antibiotic therapy and modern life support, the fatality rate of patients with sepsis has remained as high as 30%-60% worldwide [2–3]. Early identification of patients at high risk of dying from sepsis may help initiate rapid and appropriate therapeutic interventions and may have a great impact on sepsis-related morbidity and mortality. However, an accurate assessment of patients at risk for poor clinical outcomes is challenging for clinicians.

Clinical severity scores, such as the Acute Physiology and Chronic Health Evaluation (APACHEII) score and the Sequential Organ Failure Assessment (SOFA) score, have been validated for risk stratification in critical care settings [4–5]. However, clinical severity scoring tools tend to be used more in research and are not widely used in clinical decision-making. In recent years, a growing body of clinical research studies has identified blood biomarkers that may confer additional information to estimate disease progression in sepsis [6–8].

Procalcitonin (PCT), the prehormone of calcitonin, has been widely investigated in infectious diseases. Apart from its diagnostic value, PCT is also of great value for mirroring the severity of infectious diseases, such as pneumonia. In community-acquired pneumonia (CAP), PCT was shown to be a biomarker of poor outcome [9–10]. In patients suffering from ventilator-associated pneumonia (VAP), serum PCT levels could predict death and septic shock [11]. More importantly, some studies have demonstrated that PCT may confer prognostic information in sepsis[10]. An elevated PCT concentrations were reported to be strongly associated with all-cause mortality in septic patients [12]. In addition, patients at high risk of dying may suffered an persistently elevated PCT level. Thus, researches have reported that PCT non-clearance could also predict outcome of sepsis [13]. However, those studies had limited patient numbers, and the conclusions were debated. The aim of this meta-analysis was to systematically and quantitatively evaluate all available publications that assessed the prognostic accuracy of a single PCT concentration and PCT non-clearance in adult patients with sepsis and draw conclusions from these studies.

## Materials and Methods

### Search strategy and selection criteria

We systematically searched studies using PubMed, Embase, Web of Knowledge and the Cochrane Library. The search terms were as follows: (procalcitonin or PCT or "PCT clearance" or "PCT-c" or "PCT decrease" or "PCT kinetics") and (sepsis or septicemia or septicemia or septic) and (mortality or prognosis). We include articles written in English and Spanish. No publication date restrictions were applied for searching. We further reviewed the reference list of the selected articles to obtain potentially relevant articles.

Eligible studies had to have a well-defined reference standard for patients involved (sepsis or severe sepsis or septic shock) according to the criteria of the American College of Chest Physicians/Society of Critical Care Medicine [14, 15]. PCT non-clearance was defined as an persistently elevated PCT level. For studies evaluated PCT non-clearance associated with prognosis of sepsis, they should measured ΔPCT/PCT baseline, which means the relative changes in PCT—the difference between the subsequent and baseline measurement- to the baseline PCT. Studies also had to involve the collection of single PCT concentrations or PCT non-clearance data as predictors of all-cause mortality in adult (>18 years old) septic patients. Further, a 2×2 contingency table should be conducted based on those data. If multiple studies reused the same patient sample, the most recent article or the most informative article was included. For studies that assessed procalcitonin levels associated with different follow-up periods, we chose the most widely used period among the included studies.

Reviews, letters, commentaries, correspondences, case reports, conference abstracts, expert opinions, editorials and animal experiments were excluded. Articles involving pediatric patients were also excluded. Two investigators (Liu D and Su LX) independently executed the search strategy and evaluated the studies. Any disagreement was resolved by a third reviewer (Xie LX). We performed this meta-analysis according to the Preferred Reporting Items for Meta-Analyses (PRISMA) statement checklist (**S1 & S2 Files**). To improve our write-up, we have sent our paper to AJE (American Journal Experts) for edited (**S3 File**).

### Data extraction and quality assessment

The following descriptive data were extracted from the original studies: the name of the first author, publication year, the country of origin, study design, clinical setting, assay manufacturer, sample size, endpoints, the prevalence of mortality, the proportion of male patients, mean ages, the definition of PCT non-clearance, sepsis severity, cut-off point, true positives (TP), false positives (FP), false negatives (FN), true negatives (TN), sensitivity (SEN) and specificity (SPE). We contacted the corresponding authors of any study that was missing necessary data or that required clarification. We referred to the original Quality Assessment of Diagnostic Accuracy Studies (QUADAS) checklist [16] for diagnostic studies, and we revised several items to make the criteria more useful for our present meta-analysis. We evaluated the following: (1) information bias (i.e., the representativeness of the study sample and clearly described diagnostic criteria for sepsis); (2) selection bias (i.e., the recruitment of consecutive patients); (3) confusion bias (i.e., the blinding of professionals with influence on patient prognosis to PCT level); and (4) confounding bias (i.e., the exclusion of patients with comorbidities potential linked to PCT levels).

### Statistical analysis

Statistical analyses were performed using the MIDAS module in STATA version 12.0 (Stata Corporation, College Station, TX) and Meta-Disc 1.4 (XI Cochrane Colloquium, Barcelona, Spain). A *P*-value of less than 0.05 was considered statistically significant. We tabulated TP, FP, FN, and TN rates based on the effect of single PCT levels or PCT non-clearance on all-cause mortality in sepsis patients. Relative risk (RR) was used to assess the predictive value of PCT, which was pooled according to a fixed-effects or random-effects model based on DerSimonian and Lair’s method [17]. Q-test and *I* ^2^ indexes were calculated to assess inter-study heterogeneity [18–19]. Values of 25%, 50% and 75% for the *I* ^2^ test represented low, medium and high heterogeneity, respectively [20]. *I* ^2^ values of less than 50% represented acceptable between-study heterogeneity, and the fixed-effects model was selected. Otherwise, the random-effects model was selected. RRs greater than 1 indicated an increased mortality risk from exposure, and RRs less than 1 indicated a beneficial effect.

The presence of a threshold effect on the prognostic accuracy of PCT in sepsis patients was evaluated with the Spearman correlation coefficient between the logits of SEN and SPE. If no threshold effect existed, a bivariate random effects regression model [21–22] was used to calculate the pooled SEN, SPE, diagnostic odds ratio (DOR), positive likelihood ratio (PLR), and negative likelihood ratio (NLR). We also constructed a summary receiver operator characteristic (SROC) curve by plotting the individual and summary points of SEN and SPE to assess the overall diagnostic accuracy [23].

We performed subgroup analyses to explore the prognostic accuracy of PCT when restricted to different clinical settings (emergency department (ED) and intensive care unit (ICU)), studies using initial PCT levels, and studies involving patients with severe sepsis and septic shock. A univariate meta-regression analysis was performed to explore the sources of potential heterogeneity between studies. The covariates included in the analysis were as follows: the year of publication, the sample size, the prevalence of mortality, the clinical setting, the assay manufacturer, the procalcitonin testing time, the sampling method (i.e., whether studies recruited patients consecutively), and comorbidities (i.e., whether studies excluded patients with comorbidities potentially linked to PCT levels). Publication bias was tested using Deek’s funnel plot.

## Results

**Fig 1:**
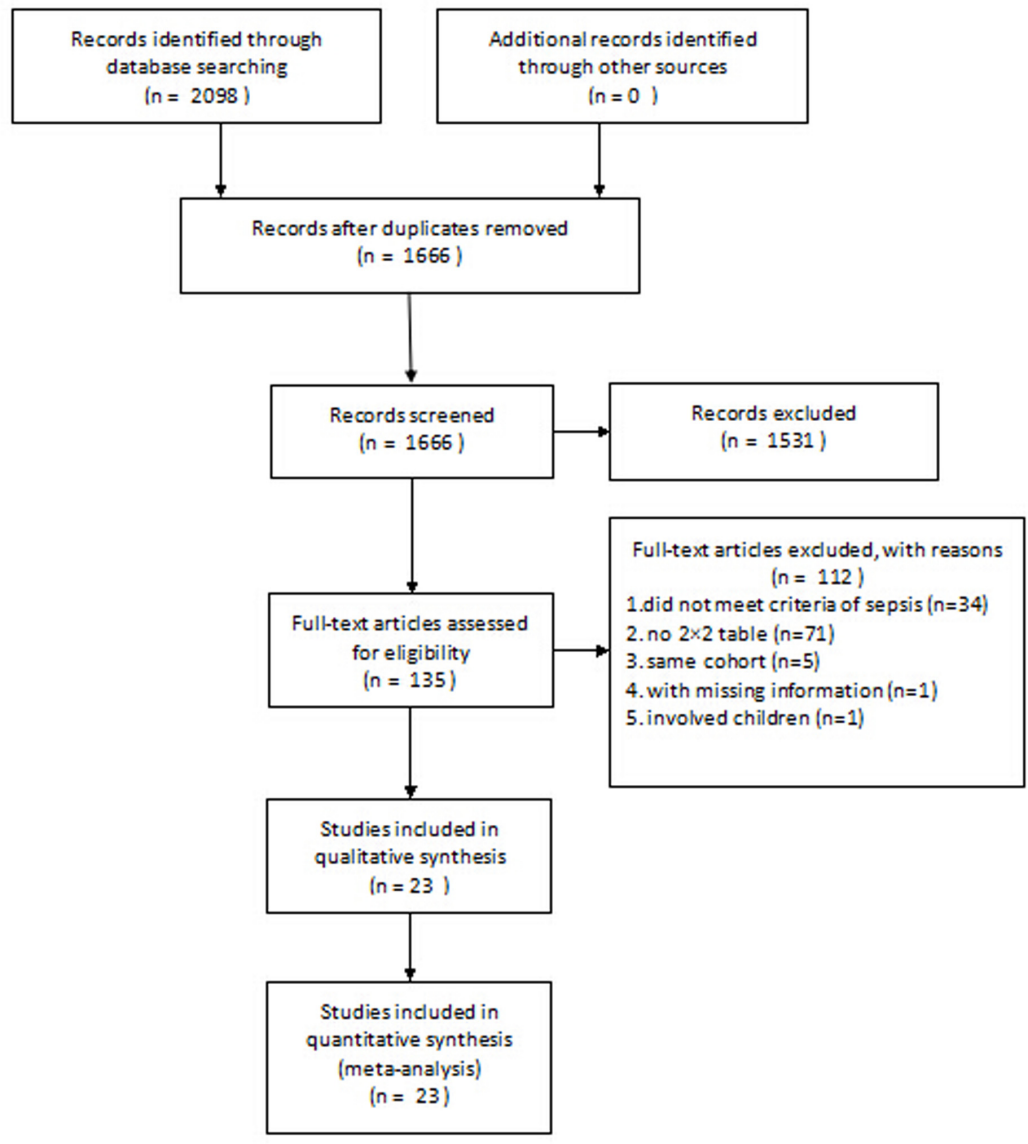
Flowchart of study selection.

Our database search retrieved 2,098 articles. We eliminated 1,963 articles for various reasons based on the title and abstract, leaving 135 studies to scrutinize with a full text review. In total, 23 studies [24–46] fulfilled our eligibility criteria and were finally included (**Fig 1**). We divided the results of two studies into two parts because investigators reported the diagnostic accuracy separately for two cohorts of patients. Thus, we analyzed 25 datasets. We did not identify any additional relevant articles in the bibliographies of original articles. The characteristics of the included studies are listed in **Table 1** and **Table 2**.

**Table 1:** Characteristics of studies associating PCT level with mortality.

Author	Year	Study design	Clinical setting	Follow-up (days)	PCT assay	Testing time	Sample size (n)	Prevalence of mortality (%)	Severity of sepsis	Cut off (ng/ml)	SEN (95% CI)	SPE (95% CI)
Adamik[24]	2000	PR	ICU	ICU mortality	Lumitest PCT	D5	41	61	Sepsis or severe sepsis	3	100	81
Meng[25]	2009	PR+CR	MICU	28-day mortality	PCT-Q	D1	86	37.2	Severe sepsis	10	75	66.7
Yin[26]	2013	PR+CR	ED	30-day mortality	VIDAS	D0	680	33.1	Sepsis, severe sepsis and septic shock	0.9	61.8	67.3
Suberviola[27]	2013	PR+CR	ICU	In-hospital mortality	KRYPTOR-PCT	D0	137	29.9	Severe sepsis and septic shock	0.83	95	14.4
Clec'h[28]	2006	PR+CR	MICU	ICU mortality	KRYPTOR-PCT	D1	36	69.4	Septic shock	6	76	72.7
Li[29]	2014	PR	ICU	28-day mortality	VIDAS	D1	102	41.2	-	10.65	76.2	81.7
Masson[30]	2014	MRCT	ICU	28-day mortality	Cobas PCT	D1	100	50	Severe sepsis and septic shock	14.27	56	60
Yaroustovsky[31]	2013	PR	ICU	28-day mortality	VIDAS	D1	81	45.7	Severe sepsis	4.76	67	62
Feng[32]	2012	PR	ICU	28-day mortality	VIDAS	D1	102	43	Sepsis and severe sepsis	8.5	67.4	73.2
Jain[33]	2014	PR+CR	MICU	28-day mortality	-	D1	54	50.9	Sepsis, severe sepsis and septic shock	7	56.6	70.9
Dahaba[34]	2006	PR	SICU	28-day mortality	Lumitest PCT	D6	69	26.1	Severe sepsis	3.2	85	89
Magrini[35]	2013	PR	ED	In-hospital mortality	VIDAS	D5	96	33.3	-	-	87	50
Savva[36]	2011	MPR+CR	ICU	28-day mortality	KRYPTOR-PCT	D1	180	28.3	Sepsis, severe sepsis and septic shock	0.92	58.8	91.5
Kenzaka[37]	2012	PR	ED	28-day mortality	PCT-Q	D1	206	9.7	Sepsis, severe sepsis and septic shock	10	55	61.3
Giamarellos-Bourboulis[38a]	2011	MPR	HW	Mortality	KRYPTOR-PCT	D1	922	17	Sepsis, severe sepsis and septic shock	0.12	88.5	27.1
Giamarellos-Bourboulis[38b]	2011	MPR	ICU	Mortality	KRYPTOR-PCT	D1	234	35.5	Sepsis, severe sepsis and septic shock	0.85	63.9	57.6

PCT = procalcitonin; ICU = intensive care unit; SICU = surgical intensive care unit; MICU = medical intensive care unit; ED = emergency department; HW = hospital ward; PR = prospective recruitment; CR = consecutive recruitment; RR = retrospective recruitment; RCT = random control trial; MPR = multiple-center prospective recruitment; MRCT = multiple-center random control trial; SEN = sensitivity; SPE = specificity; CI = confidence interval.

**Table 2:** Characteristics of studies associating PCT non-clearance with mortality.

Author	Year	Study design	Clinical setting	Follow-up (days)	PCT assay	Definition of procalcitonin non-clearance	Sample size (n)	Mortality (%)	Severity of sepsis	SEN (95% CI)	SPE (95% CI)
Tschaikowsky[39]	2011	PR+CR	SICU	28-day mortality	KRYPTOR-PCT	PCT↓ < 50% within 7d	51	33.3	Severe sepsis and septic shock	35.3	97.1
Schuetz[40a]	2013	RR+CR	ICU	ICU mortality	VIDAS	PCT↓ < 60% within 72 hr	154	29.2	Severe sepsis and septic shock	60	67
Schuetz[40b]	2013	RR+CR	ICU	ICU mortality	VIDAS	PCT↓ < 60% within 72 hr	102	17.6	Severe sepsis and septic shock	78	61
Mat Nor[41]	2014	PR	ICU	In-hospital mortality	KRYPTOR-PCT	PCT↓ < 30% within 48 hr	67	40.3	Severe sepsis	74.1	55
Ruiz-Rodriguez[42]	2012	PR	ICU	ICU mortality	Lumitest PCT	PCT↓ < 50% within 48 hr	27	66.7	Septic shock	89	72
Suberviola[43]	2012	PR	ICU	In-hospital mortality	KRYPTOR-PCT	PCT↓ < 70% within 72 hr	88	23.9	Septic shock	52.6	94.2
Karlsson[44]	2010	PR+CR	ICU	In-hospital mortality	Cobas PCT	PCT↓ < 50% within 72 hr	242	24.2	Severe sepsis	88.7	27.8
Garcia de Guadiana-Romualdo[45]	2014	PR	ICU	In-hospital mortality	Cobas PCT	PCT↓ < 40% within 48 hr	100	28	Severe sepsis and septic shock	64.3	62.5
Guan[46]	2011	PR	ICU	Mortality	Lumitest PCT	PCT↓ < 25% within 5d	37	32.4	Sepsis, severe sepsis and septic shock	100	100

PCT = procalcitonin; ICU = intensive care unit; SICU = surgical intensive care unit; ED = emergency department; HW = hospital ward; PR = prospective recruitment; CR = consecutive recruitment; RR = retrospective recruitment; RCT = random control trial; MPR = multiple-center prospective recruitment; MRCT = multiple-center random control trial; SEN = sensitivity; SPE = specificity; CI = confidence interval.

### Characteristics of included studies

The included studies were published from 2000 to 2014. Thirteen studies [24, 27, 28, 30–31, 35–36, 38–39, 42–45] were conducted in Europe, eight [25–26, 29, 32–33, 37, 41, 46] were conducted in Asia, one [40] was conducted in America, and one [34] in was conducted in Australia. With one exception [45], all studies were published in English. The mean age of patients varied between 45 and 75.8 years, and the proportion of men ranged from 44.8 to 70.4%. Twelve studies [25, 27, 30–31, 34, 39–45] included only patients with severe sepsis or septic shock. The most frequent source of sepsis was pulmonary infection. Three studies [26, 35, 37] were performed in EDs, one [38a] was performed in a hospital ward, and the remaining studies were performed in an ICU. With respect to admission category characteristics, three studies [25, 28, 33] involved only medical patients, two [34, 39] involved only surgical patients, and the remaining studies involved both medical and surgical patients. Fifteen studies [24–38] evaluated the effect of single PCT concentrations on all-cause mortality in sepsis patients. Among these studies, three [24, 34–35] measured PCT level on the fifth or sixth day after admission and twelve collected blood samples within 24 h of sepsis diagnosis. Eight studies [39–46] evaluated PCT clearance. Follow-up periods differed across studies, including 28 days [25, 29–33], 30 days [26], ICU stays [24, 28] and in-hospital stays [27].

### Study quality and publication bias

**Fig 2:**
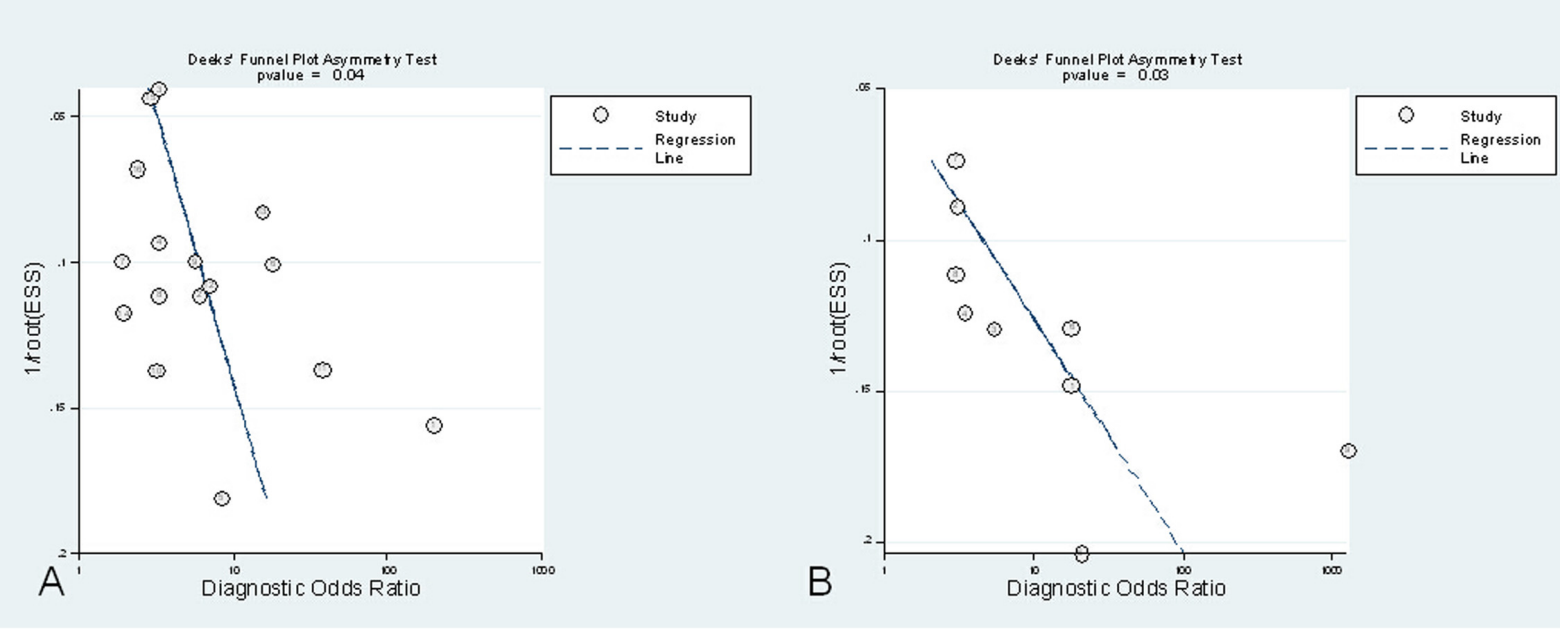
Deek’s funnel plot asymmetry test for publication bias (A. For single procalcitonin concentration; B. For procalcitonin non-clearance). Potential publication bias exists (P<0.05).

All studies included a representative sample of patients who underwent PCT testing in practice and clearly described the diagnostic criteria for sepsis. Nine studies [25–28, 33, 36, 39–40, 44] included consecutive patients. Three studies [35, 42, 46] mentioned the blinding of professionals who influenced patient prognosis to PCT level. Four studies [24, 29, 33, 35] excluded patients with comorbidities potentially linked to PCT levels, such as end-organ damage and autoimmune diseases. The Deek’s funnel plot of the included studies suggested the presence of publication bias (**Fig 2A and 2B**).

### Data synthesis and meta-analysis

#### Analysis of the association of PCT concentration with mortality

**Fig 3:**
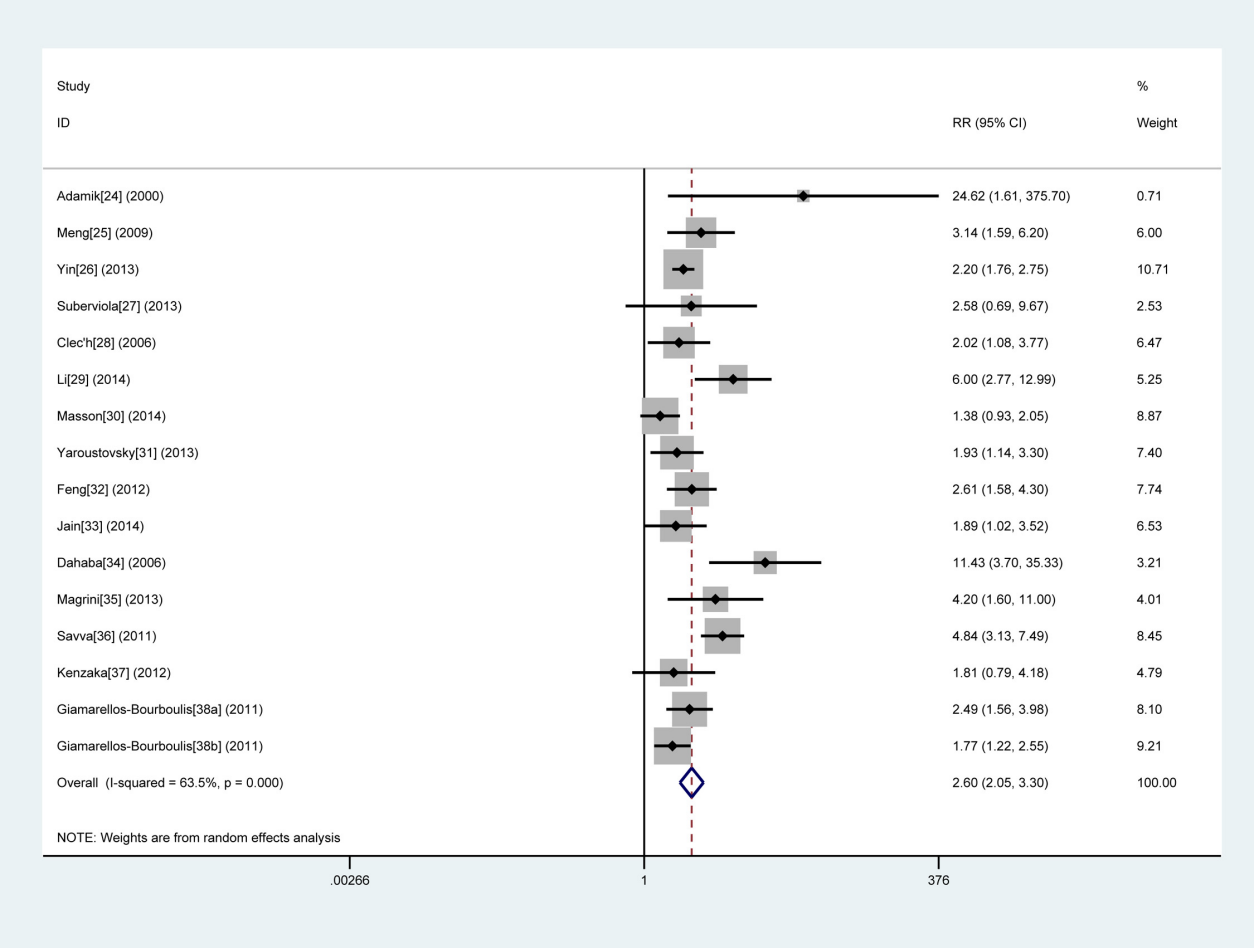
Forest plot of procalcitonin concentration to predict mortality in sepsis. The overall pooled RR was 2.60 (95% CI, 2.05–3.30).

Sixteen studies [24–38] with 3126 patients were included in this group. All studies showed that an elevated PCT level was associated with a higher risk of death, with RR ranging from 1.38 to 24.62. Because of the substantial heterogeneity between studies (*I* ^2^ = 63.5%), a random-effects model was used to pool RR estimates. The pooled RR was 2.60 (95% CI, 2.05–3.30) (**Fig 3**).

**Fig 4:**
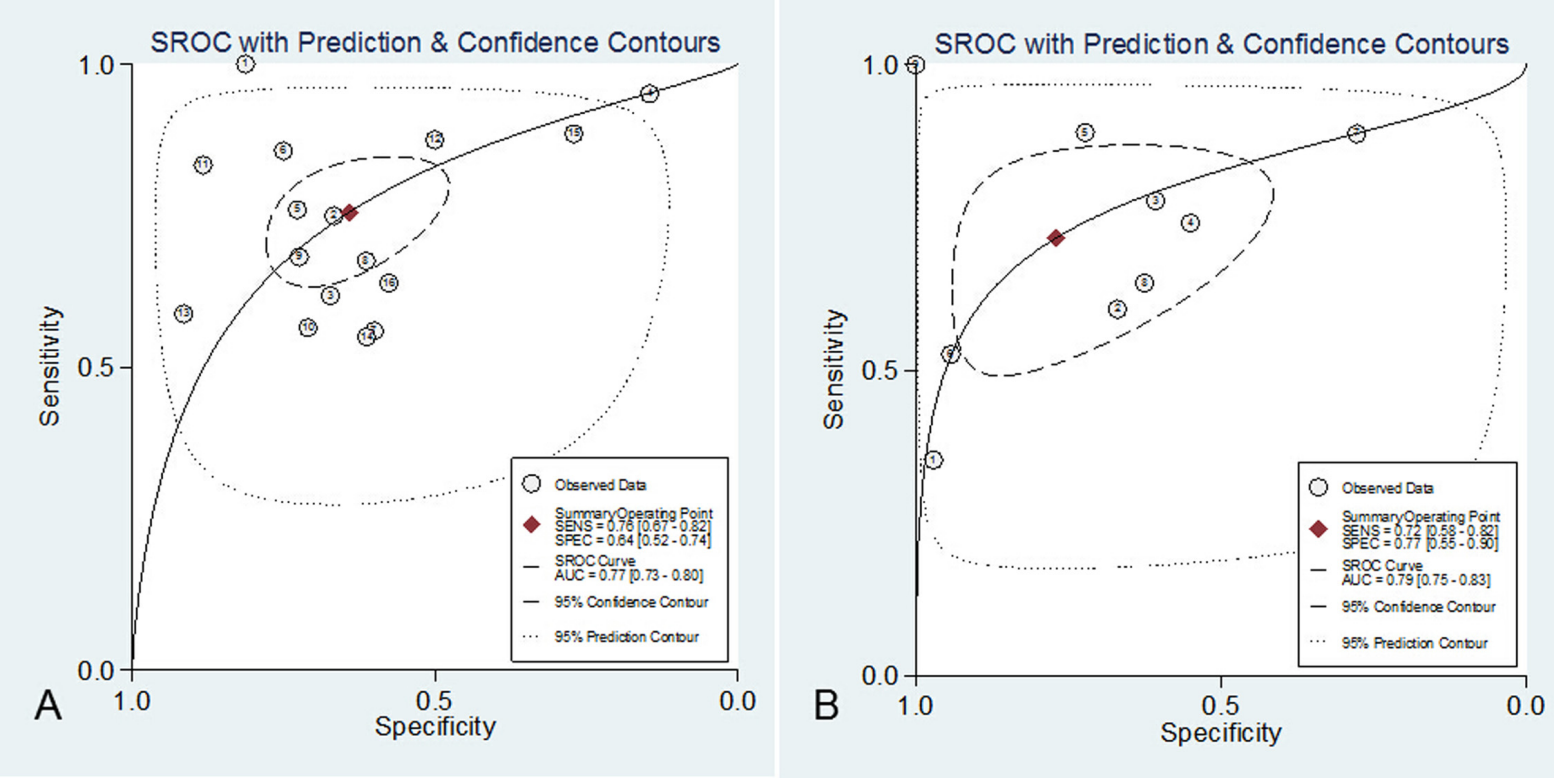
Summary receiver operating characteristic graph of the included studies (A. For single procalcitonin concentration; B. For procalcitonin non-clearance).

No statistically significant difference was observed when exploring for threshold effect (Spearman correlation coefficient = 0.062; *P* = 0.820). The pooled SEN and SPE were 0.76 (95% CI, 0.67–0.82) and 0.64 (95% CI, 0.52–0.74), respectively (**S1 Fig**). The PLR and NLR were 2.1 (95% CI, 1.6–2.8) and 0.38 (95% CI, 0.29–0.51), respectively. The DOR was 6 (95% CI, 3–9). The overall area under the SROC curve was 0.77 (95% CI, 0.73–0.80) (**Fig 4A**).

A univariate meta-regression analysis was performed to explore the sources of potential heterogeneity between studies. The year of publication, the sample size, the prevalence of mortality, the clinical setting, the assay manufacturer, the PCT testing time, the sampling method (i.e., whether studies recruited patients consecutively), and comorbidities (i.e., whether studies excluded patients with comorbidities potentially linked to PCT levels) were included in the analysis. The results indicated that only PCT testing time was statistically significant for heterogeneity (*P* = 0.020). The initial PCT level was of limited prognostic value in patients with sepsis. Subgroup analyses are shown in **Table 3**.

**Table 3:** Subgroup analysis.

Variables	No. of studies	No. of patients	SEN (95% CI)	SPE (95% CI)	DOR (95% CI)	PLR (95% CI)	NLR (95% CI)	AUC (95% CI)	Test for heterogeneity (*I*2)	Deek's funnel test (*p* value)
Overall	16	3126	0.76(0.67–0.82)	0.64(0.52–0.74)	6(3–9)	2.1(1.6–2.8)	0.38(0.29–0.51)	0.77(0.73–0.80)	63.5	0.04
Initial PCT concentration	13	2920	0.72(0.63–0.79)	0.62(0.49–0.73)	4(3–6)	1.9(1.4–2.4)	0.46(0.37–0.56)	0.73(0.69–0.77)	57.5	0.29
ICU patients	12	1222	0.76(0.65–0.84)	0.69(0.55–0.80)	7(4–13)	2.4(1.7–3.6)	0.35(0.24–0.51)	0.79(0.75–0.82)	72.1	0.12
Severe sepsis/septic shock	6	509	0.77(0.62–0.87)	0.61(0.38–0.80)	5(2–11)	1.9(1.2–3.2)	0.39(0.25–0.61)	0.76(0.73–0.80)	67.3	0.17

PCT = procalcitonin; ICU = intensive care unit; ED = emergency department; SEN = sensitivity; SPE = specificity; DOR = diagnostic odds ratio; PLR = positive likelihood ratio; NLR = negative likelihood ratio; AUC = area under the curve; CI = confidence interval.

#### Analysis of the effect of PCT non-clearance on mortality

**Fig 5:**
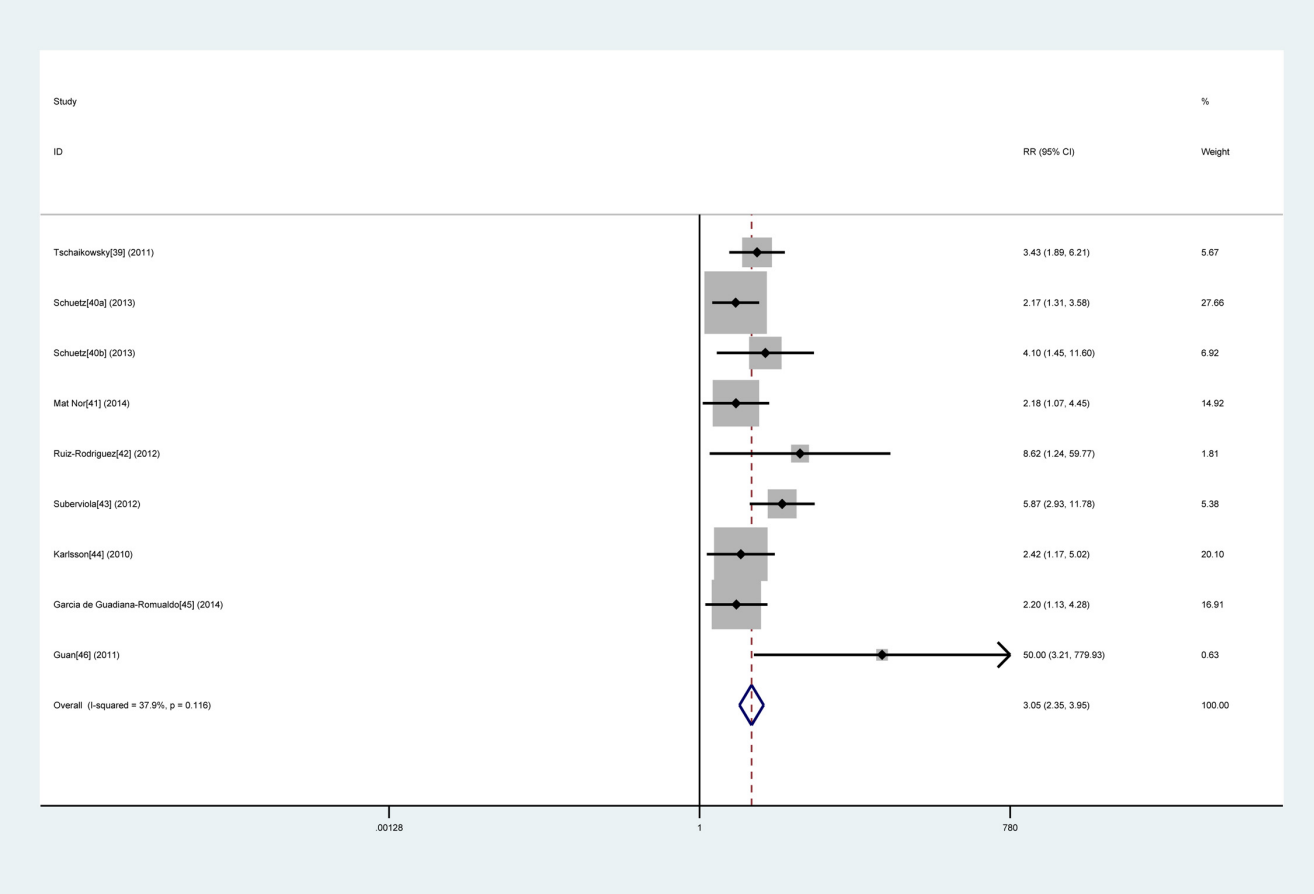
Forest plot of procalcitonin non-clearance to predict mortality in sepsis. The overall pooled RR was 3.05 (95% CI, 2.35–3.95).

Nine studies [39–46] with 868 patients were included in this group. Because the heterogeneity between studies was acceptable (*I* ^2^ = 37.9%), a fixed-effects model was used to pool RR estimates. The pooled RR for mortality was 3.05 (95% CI, 2.35–3.95) (**Fig 5**).

No statistically significant differences were observed when exploring the threshold effect (Spearman correlation coefficient = 0.133; *P* = 0.732). The pooled SEN and SPE were 0.72 (95% CI, 0.58–0.82) and 0.77 (95% CI, 0.55–0.90), respectively (**S2 Fig**). The PLR and NLR were 3.1 (95% CI, 1.5–6.3) and 0.37 (95% CI, 0.25–0.55), respectively. The DOR was 8 (95% CI, 3–22). The overall area under the SROC curve was 0.79 (95% CI, 0.75–0.83) (**Fig 4B**).

## Discussion

In this meta-analysis, we first determined that both single PCT concentrations and PCT non-clearance were strongly associated with all-cause mortality in septic patients. Our evidence may confer additional information for the clinical use of PCT apart from diagnosing infection [47] and helping guide therapeutic decision-making [48].

We further identified that PCT non-clearance could predict sepsis mortality. The pooled RR for mortality was 3.05 (95% CI, 2.35–3.95). The overall area under the SROC curve was 0.79 (95% CI, 0.75–0.83). We evaluated the prognostic performance of PCT, and the results showed that the diagnostic performance of both a single PCT concentration and PCT clearance is moderate for predicting sepsis mortality.

The implementation of the appropriate therapeutic interventions appeared to be more significant when initiated rapidly at the time of the patient’s arrival. Delayed resuscitation has been found to be significantly associated with a risk of death [49–50]. The prognostic value of biomarkers have been widely investigated in other meta-analyses [51, 52]. However, those meta-analyses were not immune to unexplained heterogeneity and had a limited number of patients. In our research, PCT non-clearance has emerged as an ideal index to predict prognosis in sepsis. The overall area under the SROC curve was 0.79 (95% CI, 0.75–0.83), which was greater than the results of published meta-analyses of troponins [51] and lactate clearance [52]. In addition, the heterogeneity between studies was acceptable (*I* ^2^ = 37.9%), which showed our results were reliable. The initial PCT level was of limited prognostic value in patients with sepsis. The pooled SEN and SPE were 0.72 (95% CI, 0.63–0.79) and 0.62 (95% CI, 0.49–0.73), respectively. The overall area under the SROC curve was only 0.73 (95% CI, 0.69–0.77).

Our study has several limitations. First, we failed to assess the diagnostic accuracy of PCT for predicting death in ED patients because of the limited number of available studies. Thus, our results probably cannot be applied to ED patients. Second, also owing to the limited number of available studies, we could not perform subgroup analyses based on different admission categories and sites of infection. Third, we could not determine the optimized cut-off value because we failed to obtain the raw data (the procalcitonin level in each patient) from each original article to map out the ROC curve. We have tried to contact the corresponding authors to obtain the data, but has been difficult to acquire the procalcitonin level of each patients in each trail. And also, we could not conclude the optimal definition of PCT non-clearance required for accurate risk assessment.

Sepsis is a complex pathophysiological process rather than a specific syndrome. Thus far, no ideal biomarker has demonstrated sufficient SEN and SPE to provide clinical utility for predicting sepsis mortality [53]. Clinicians need to provide a more comprehensive evaluation of individual patient conditions. Future studies should highlight the combination of procalcitonin with other clinical indexes as part of an overall assessment of sepsis prognosis rather than adopting a biomarker-based approach to the prediction of sepsis mortality. The combination of PCT and other clinical indexes may provide valuable information to assist clinicians in identifying patients at high risk of dying from sepsis. Several studies [8, 22] showed that PCT concentrations were related to APACHEII and SOFA scores. Of the studies included in this meta-analysis, Suberviola [20] demonstrated an improved prognostic value when PCT was combined with the APACHEII score. Further studies should be performed to help determine the optimal cut-off point and definition for PCT non-clearance required for accurate risk assessment.

## Conclusions

We found that elevated PCT levels and PCT non-clearance were associated with a higher risk of death in patients with sepsis. However, PCT may not be useful as a single index for assessing prognosis because of its moderate diagnostic accuracy, though it may be useful in combination with patients’ overall conditions and other clinical indexes. Further studies are needed to define the optimal cut-off point and a definition of PCT non-clearance required for accurate risk assessment.

## Supporting Information

S1 File PRISMA 2009 Flow Diagram. (DOC)

S2 File PRISMA 2009 checklist. (DOC)

S3 File AJE_Edited_XJQYC7J3_modified_manuscript_Prognosis_value_of_procalcitonin_in_sepsis_a_systematic_review_and_meta_analysis_SE_QCE. (DOC)

S4 File Collection of tables in the manuscript. (DOC)

S1 Fig Forest plot of the sensitivity and specificity of procalcitonin concentration for predicting mortality in sepsis. (TIF)

S2 Fig Forrest plot of the sensitivity and specificity of procalcitonin non-clearance for predicting mortality in sepsis. (TIF)
