# Supplementary material for: Prognostic Value of Procalcitonin in Adult Patients with Sepsis: A Systematic Review and Meta-Analysis
Source: PLoS One. 2015 Jun 15;10(6):e0129450. doi: 10.1371/journal.pone.0129450 (PMC4468164; doi:10.1371/journal.pone.0129450)
Supplement: S1 File — (DOC) [file pone.0129450.s001.doc]

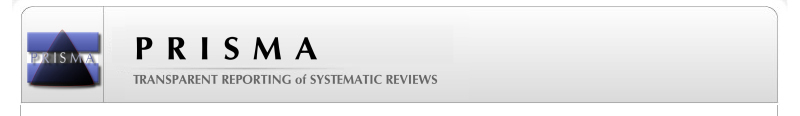
**PRISMA 2009 Flow Diagram**

**Screening**

**Included**

**Eligibility**

**Identification**

Records identified through database searching
(n = 2098 )

Additional records identified through other sources
(n = 0 )

Records after duplicates removed
(n = 1666 )

Records screened
(n = 1666 )

Records excluded
(n = 1531 )

Full-text articles assessed for eligibility
(n = 135 )

Full-text articles excluded, with reasons
(n = 112 )

1.did not meet criteria of sepsis (n=34)

2. no 2×2 table (n=70)

3. measured procacitonin increase (n=1)

4. same cohort (n=5)

5. with missing information (n=1)

6. involved children (n=1)

Studies included in qualitative synthesis
(n = 23 )

Studies included in quantitative synthesis (meta-analysis)
(n = 23 )
