# Supplementary material for: Prognostic Value of Procalcitonin in Adult Patients with Sepsis: A Systematic Review and Meta-Analysis
Source: PLoS One. 2015 Jun 15;10(6):e0129450. doi: 10.1371/journal.pone.0129450 (PMC4468164; doi:10.1371/journal.pone.0129450)
Supplement: S2 File — (DOC) [file pone.0129450.s002.doc]

| **Section/topic** | **#** | **Checklist item** | **Reported on page #** |
| --- | --- | --- | --- |
| **TITLE** | | |  |
| Title | 1 | Prognosis value of procalcitonin in sepsis: a systematic review and meta-analysis | 1 |
| **ABSTRACT** | | |  |
| Structured summary | 2 | The purpose of the present meta-analysis is to explore the diagnostic accuracy of single PCT concentration and PCT non-clearance to predict all-cause mortality in sepsis.We searched PubMed, Embase, Web of Knowledge and Cochrane databases. Articles written in English were included. A 2 × 2 contingency table could be conducted based on all-cause mortality and PCT level or PCT non-clearance in septic patients. Two authors independently judged studies and extracted data. The diagnostic value of PCT in predicting prognosis was determined using a bivariate meta-analysis model. We used the Q-test and *I*^2^ index to test heterogeneity. 23 studies with a total of 3,994 patients were included. An elevated PCT level was associated with higher risk of death. The pooled RR was 2.60 (95% CI 2.05-3.30), random-effects model was used (I-squared=63.5%). Measured timing were statistically significant for heterogeneity (*P* =0.020). Initial PCT was of limited prognostic value in patients with sepsis. PCT non-clearance was a factor of death in sepsis. The pooled RR was 3.05 (95%CI, 2.35-3.95), fixed-effects model was used (I-squared=37.9%). The diagnostic performance of single PCT concentration and PCT non-clearance are both moderate to predict mortality in sepsis. | 2 |
| **INTRODUCTION** | | |  |
| Rationale | 3 | Accurately assessing septic patients at risk of poor outcome is challenging for clinicians. Procalcitonin (PCT) has been widely investigated for its prognostic value in septic patients. However, the results are conflicting. | 2-3 |
| Objectives | 4 | We aim to explore the diagnostic accuracy of single PCT concentration and PCT non-clearance to predict all-cause mortality in sepsis. | 3 |
| **METHODS** | | |  |
| Protocol and registration | 5 | N/A |  |
| Eligibility criteria | 6 | Eligible studies had a well-defined reference standard for patients diagnosed with sepsis. A 2×2 contingency table should be constructed based on single procaocitonin concentration or procalcitonin non-clearance correlated with all-cause mortality in adult (>18 years old) patients with sepsis. | 4 |
| Information sources | 7 | A systematic search was performed to identify studies using PubMed, Embase, Web of Knowledge and the Cochrane Library. We contacted to correspondence author if the information needed were not exist in articles. | 4 |
| Search | 8 | Searching terms were as follows: (procalcitonin or PCT or "PCT clearance" or "PCT-c" or "PCT decrease" or "PCT kinetics") and (sepsis or septicemia or septicaemia or septic) and (mortality or prognosis). | 3 |
| Study selection | 9 | Reviews, letters, commentaries, correspondences, case reports, conference abstracts, expert opinions, editorials and animal experiments were excluded. Articles involving pediatric patients were excluded. | 4 |
| Data collection process | 10 | Two investigators independently performed the search strategy and collected data. Any disagreement was resolved by a third opinion. | 4 |
| Data items | 11 | The following descriptive data were extracted from the original studies: name of the first author, publication year, country of origin, study design, clinical setting, assay manufacturer, sample size, endpoints, prevalence of mortality, the proportion of male, mean ages, definition of PCT non-clearance, severity of sepsis, cut-off point, true positive (TP), false positive (FP), false negative (FN), true negative (TN), sensitivity (SEN) and specificity (SPE) of the data. | 4 |
| Risk of bias in individual studies | 12 | We evaluate (1) information bias: if the spectrum of included patients was representative of the patients who will undergo testing of procalcitonin in practice, and if the selection criteria of sepsis was clearly described; (2) selection bias: if the study included consecutive patients; (3) confusion bias: if the professionals who influenced the patient prognosis were blinded to PCT level; (4) confounding bias: if the study excluded patients with comorbidities susceptible to the influence PCT levels, such as end-organ damage and autoimmune diseases. | 4,5 |
| Summary measures | 13 | We calculated the pooled relative risk (RR), the pooled sensitivity (SEN), specificity (SPE), diagnostic odds ratio (DOR), positive likelihood ratio (PLR), and negative likelihood ratio (NLR) and the summary receiver operator characteristic (SROC) curve. | 5,6 |
| Synthesis of results | 14 | The diagnostic value of procalcitonin in predicting prognosis was determined using a bivariate meta-analysis model. We used the Q-test and *I*^2^ index to test heterogeneity. | 6 |

Page 1 of 2

| **Section/topic** | **#** | **Checklist item** | **Reported on page #** |
| --- | --- | --- | --- |
| Risk of bias across studies | 15 | Publication bias was tested by Deek’s funnel plot. | 6 |
| Additional analyses | 16 | Subgroup analysis and univariate meta-regression analysis were performed to explore the sources of potential heterogeneity between-studies. | 6 |
| **RESULTS** | | |  |
| Study selection | 17 | Our database search retrieved 2098 articles. 2951 were eliminated for various reasons based on the title and abstract, leaving 135 studies scrutinized for a full text review. 23 studies fulfilled our eligibility criteria and were finally included. Because in two studies investigators reported diagnostic accuracy separately on two cohorts of patients, the study was divided into two parts, thus we analyzed 25 datasets. We did not identify any more relevant articles in the bibliographies of original articles. | 6 |
| Study characteristics | 18 | All studies but one were published in English. The mean age of the patients varied between 45 and 75.8 years and the proportion of men ranged from 44.8 to 70.4. Twelve studies only included patients with severe sepsis or sepsis shock. The most frequent source of sepsis was pulmonary infection. Three studies were performed in emergency department (ED); one were performed in hospital ward; others were in ICU. Fifteen studies evaluated single PCT concentration associated with all-cause mortality in sepsis. Among them, three studies measured PCT level on the fifth or sixth day after admission, whereas others collected blood samples within 24h after patients diagnosed of sepsis. Eight studies evaluated PCT clearance. Follow-up periods differed across studies, including 28 days; 30 days; ICU stay and in-hospital stay. | 6,7 |
| Risk of bias within studies | 19 | All studies included patients representative of the patients who will undergo testing of PCT in practice and clearly described the selection criteria of sepsis. 50% of the studies included consecutive patients. 10% of the studies mentioned the professionals who influenced the patient prognosis were blinded to PCT level. | Figure 2 |
| Results of individual studies | 20 | In all studies, an elevated PCT level and PCT non-clearance turned out to be a risk factor of mortality in CAP | 7,8 |
| Synthesis of results | 21 | Single PCT concentration and PCT non-clearance is strongly associated with all-cause mortality in septic patients.The pooled RR was 2.60 and 3.05,respectively.Initial PCT was of limited prognostic value in patients with sepsis. | 7 |
| Risk of bias across studies | 22 | Deek’s Funnel plot showed potential no publication bias exists | 7 |
| Additional analysis | 23 | Meta-regression showed that measured timing were statistically significant for heterogeneity (*P* =0.020). Initial PCT was of limited prognostic value in patients with sepsis. | 8 |
| **DISCUSSION** | | |  |
| Summary of evidence | 24 | We identified that single PCT concentration and PCT non-clearance are both strongly associated with all-cause mortality in septic patients.The diagnostic performance of single PCT concentration and PCT non-clearance are both moderate to predict mortality in sepsis.Initial PCT was of limited prognostic value in patients with sepsis. | 8,9 |
| Limitations | 25 | First, we failed to assess the diagnostic accuracy of PCT to predict death in ED because of limited number of studies.Second, also because of a limited number of studies included, we could not perform subgroup analysis based on different admission category and different sites of infection. Third, we could not determine the optimized cut-off value of single PCT because we failed to obtain the raw data to map out the ROC curve. Further, we could not conclude the optimal definition of PCT non-clearance for risk assessment. | 9 |
| Conclusions | 26 | We confirmed that single PCT concentration and PCT non-clearance are both strongly associated with all-cause mortality in septic patients. | 10 |
| **FUNDING** | | |  |
| Funding | 27 | N/A |  |

*From:*  Moher D, Liberati A, Tetzlaff J, Altman DG, The PRISMA Group (2009). Preferred Reporting Items for Systematic Reviews and Meta-Analyses: The PRISMA Statement. PLoS Med 6(6): e1000097. doi:10.1371/journal.pmed1000097

For more information, visit: **www.prisma-statement.org**.

Page 2 of 2
