# Supplementary material for: Prognostic Value of Procalcitonin in Adult Patients with Sepsis: A Systematic Review and Meta-Analysis
Source: PLoS One. 2015 Jun 15;10(6):e0129450. doi: 10.1371/journal.pone.0129450 (PMC4468164; doi:10.1371/journal.pone.0129450)
Supplement: S3 File — (DOC) [file pone.0129450.s003.doc]

**Prognostic value of procalcitonin in adult patients with sepsis: a systematic review and meta-analysis**

Dan Liu^1, 2, 3¶^, Longxiang Su^4¶^, Gencheng Han^5^, Peng Yan^1^, Lixin Xie^1*^

^1^ Department of Pulmonary & Critical Care Medicine, Chinese PLA General Hospital, 28 Fuxing Road, Beijing, 100853, China

^2^ Medical School, Nankai University, 94 Weijin Road, Tianjin, 300071, China

^3^ Department of Respiratory Medicine, Tianjin Medical University General Hospital, Tianjin, 300070, China

^4^ Department of Critical Care Medicine, Peking Union Medical College Hospital, Peking Union Medical College & Chinese Academy of Medical Sciences, Beijing, 100005, China

^5^ Laboratory of Immunology, Institute of Basic Medical Sciences, Beijing, 100850, China

^*^Corresponding author

Email: xielx@263.net

^¶^ These authors contributed equally to this work.

**Abstract**

**Introduction**: Procalcitonin (PCT) has been widely investigated for its prognostic value in septic patients. However, studies have produced conflicting results. The purpose of the present meta-analysis is to explore the diagnostic accuracy of a single PCT concentration and PCT non-clearance in predicting all-cause sepsis mortality.

**Methods**: We searched PubMed, Embase, Web of Knowledge and the Cochrane Library. Articles written in English were included. A 2 × 2 contingency table was constructed based on all-cause mortality and PCT level or PCT non-clearance in septic patients. Two authors independently evaluated study eligibility and extracted data. The diagnostic value of PCT in predicting prognosis was determined using a bivariate meta-analysis model. We used the Q-test and *I*^2^ index to test heterogeneity.

**Results**: Twenty-three studies with 3,994 patients were included. An elevated PCT level was associated with a higher risk of death. The pooled relative risk (RR) was 2.60 (95% confidence interval (CI), 2.05-3.30) using a random-effects model (*I*^2^=63.5%). The overall area under the summary receiver operator characteristic (SROC) curve was 0.77 (95% CI, 0.73–0.80), with a sensitivity and specificity of 0.76 (95% CI, 0.67–0.82) and 0.64 (95% CI, 0.52–0.74), respectively. There was significant evidence of heterogeneity for the PCT testing time (*P*=0.020). Initial PCT values were of limited prognostic value in patients with sepsis. PCT non-clearance was a prognostic factor of death in patients with sepsis. The pooled RR was 3.05 (95% CI, 2.35-3.95) using a fixed-effects model (*I*^2^=37.9%). The overall area under the SROC curve was 0.79 (95% CI, 0.75–0.83), with a sensitivity and specificity of 0.72 (95% CI, 0.58–0.82) and 0.77 (95% CI, 0.55–0.90), respectively.

**Conclusions**: Elevated PCT concentrations and PCT non-clearance are strongly associated with all-cause mortality in septic patients. Further studies are needed to define the optimal cut-off point and the optimal definition of PCT non-clearance for accurate risk assessment.

**Key words**: Procalcitonin; Sepsis; Mortality; Meta-analysis

**1. Introduction**

Sepsis is a life-threatening condition that arises when the body’s response to an infection injures its own tissues and organs [1]. Despite advances in antibiotic therapy and modern life support, the fatality rate of patients with sepsis has remained as high as 30%-60% worldwide [2-3]. Early identification of patients at high risk of dying from sepsis may help initiate rapid and appropriate therapeutic interventions and may have a great impact on sepsis-related morbidity and mortality. However, an accurate assessment of patients at risk for poor clinical outcomes is challenging for clinicians.

Clinical severity scores, such as the Acute Physiology and Chronic Health Evaluation (APACHEII) score and the Sequential Organ Failure Assessment (SOFA) score, have been validated for risk stratification in critical care settings [4-5]. However, clinical severity scoring tools tend to be used more in research and are not widely used in clinical decision-making. In recent years, a growing body of clinical research studies has identified blood biomarkers that may confer additional information to estimate disease progression in sepsis [6-8].

Procalcitonin (PCT), the prehormone of calcitonin, has been widely investigated in infectious diseases. Apart from its diagnostic value, PCT is also of great value for mirroring the severity of infectious diseases, such as pneumonia. In community-acquired pneumonia (CAP), PCT was shown to be a biomarker of poor outcome [9]. Further, some studies have demonstrated that PCT may confer important prognostic information when combined with clinical scores [10]. In patients suffering from ventilator-associated pneumonia (VAP), serum PCT levels could predict survival and septic shock [11]. Several studies have been performed to evaluate the potential role of procalcitonin level as well as procalcitonin kinetics to predict prognosis of sepsis [12-13]. However, those studies had limited patient numbers, and the conclusions were debated. The aim of this meta-analysis was to systematically and quantitatively evaluate all available publications that assessed the prognostic accuracy of a single PCT concentration and PCT non-clearance in adult patients with sepsis and draw conclusions from these studies.

**2. Methods**

**2.1 Search strategy and selection criteria**

We systematically searched studies using PubMed, Embase, Web of Knowledge and the Cochrane Library. The search terms were as follows: (procalcitonin or PCT or "PCT clearance" or "PCT-c" or "PCT decrease" or "PCT kinetics") and (sepsis or septicemia or septicemia or septic) and (mortality or prognosis). We include articles written in English and Spanish. No publication date restrictions were applied for searching. We further reviewed the reference list of the selected articles to obtain potentially relevant articles.

Eligible studies had to have a well-defined reference standard for patients involved (sepsis or severe sepsis or septic shock) according to the criteria of the American College of Chest Physicians/Society of Critical Care Medicine [14, 15]. Studies also had to involve the collection of single PCT concentrations or PCT clearance data as predictors of all-cause mortality in adult (>18 years old) septic patients and include a 2×2 contingency table of those data. If multiple studies reused the same patient sample, the most recent article or the most informative article was included. For studies that assessed procalcitonin levels associated with different follow-up periods, we chose the most widely used period among the included studies.

Reviews, letters, commentaries, correspondences, case reports, conference abstracts, expert opinions, editorials and animal experiments were excluded. Articles involving pediatric patients were also excluded. Two investigators (Liu D and Su LX) independently executed the search strategy and evaluated the studies. Any disagreement was resolved by a third reviewer (Xie LX).

**2.2 Data extraction and quality assessment**

The following descriptive data were extracted from the original studies: the name of the first author, publication year, the country of origin, study design, clinical setting, assay manufacturer, sample size, endpoints, the prevalence of mortality, the proportion of male patients, mean ages, the definition of PCT non-clearance, sepsis severity, cut-off point, true positives (TP), false positives (FP), false negatives (FN), true negatives (TN), sensitivity (SEN) and specificity (SPE). We contacted the corresponding authors of any study that was missing necessary data or that required clarification. We referred to the original Quality Assessment of Diagnostic Accuracy Studies (QUADAS) checklist [16] for diagnostic studies, and we revised several items to make the criteria more useful for our present meta-analysis. We evaluated the following: (1) information bias (i.e., the representativeness of the study sample and clearly described diagnostic criteria for sepsis); (2) selection bias (i.e., the recruitment of consecutive patients); (3) confusion bias (i.e., the blinding of professionals with influence on patient prognosis to PCT level); and (4) confounding bias (i.e., the exclusion of patients with comorbidities potential linked to PCT levels).

**2.3 Statistical analysis**

Statistical analyses were performed using the MIDAS module in STATA version 12.0 (Stata Corporation, College Station, TX) and Meta-Disc 1.4 (XI Cochrane Colloquium, Barcelona, Spain). A *P*-value of less than 0.05 was considered statistically significant. We tabulated TP, FP, FN, and TN rates based on the effect of single PCT levels or PCT non-clearance on all-cause mortality in sepsis patients. Relative risk (RR) was used to assess the predictive value of PCT, which was pooled according to a fixed-effects or random-effects model based on DerSimonian and Lair’s method [17]. Q-test and *I*^2^ indexes were calculated to assess inter-study heterogeneity [18-19]. Values of 25%, 50% and 75% for the *I*^2^ test represented low, medium and high heterogeneity, respectively [20]. *I*^2^ values of less than 50% represented acceptable between-study heterogeneity, and the fixed-effects model was selected. Otherwise, the random-effects model was selected. RRs greater than 1 indicated an increased mortality risk from exposure, and RRs less than 1 indicated a beneficial effect.

The presence of a threshold effect on the prognostic accuracy of PCT in sepsis patients was evaluated with the Spearman correlation coefficient between the logits of SEN and SPE. If no threshold effect existed, a bivariate random effects regression model [21-22] was used to calculate the pooled SEN, SPE, diagnostic odds ratio (DOR), positive likelihood ratio (PLR), and negative likelihood ratio (NLR). We also constructed a summary receiver operator characteristic (SROC) curve by plotting the individual and summary points of SEN and SPE to assess the overall diagnostic accuracy [23].

We performed subgroup analyses to explore the prognostic accuracy of PCT when restricted to different clinical settings (emergency department (ED) and intensive care unit (ICU)), studies using initial PCT levels, and studies involving patients with severe sepsis and septic shock. A univariate meta-regression analysis was performed to explore the sources of potential heterogeneity between studies. The covariates included in the analysis were as follows: the year of publication, the sample size, the prevalence of mortality, the clinical setting, the assay manufacturer, the procalcitonin testing time, the sampling method (i.e., whether studies recruited patients consecutively), and comorbidities (i.e., whether studies excluded patients with comorbidities potentially linked to PCT levels). Publication bias was tested using Deek’s funnel plot.

**3. Results**

Our database search retrieved 2,098 articles. We eliminated 1,963 articles for various reasons based on the title and abstract, leaving 135 studies to scrutinize with a full text review. In total, 23 studies fulfilled our eligibility criteria and were finally included (**Fig. 1**). We divided the results of two studies into two parts because investigators reported the diagnostic accuracy separately for two cohorts of patients. Thus, we analyzed 25 datasets. We did not identify any additional relevant articles in the bibliographies of original articles. The characteristics of the included studies are listed in **Table 1** and **Table 2**.

**3.1 Characteristics of included studies**

The included studies were published from 2000 to 2014. Thirteen studies [24, 27, 28, 30-31, 35-36, 38-39, 42-45] were conducted in Europe, eight [25-26, 29, 32-33, 37, 41, 46] were conducted in Asia, one [40] was conducted in America, and one [34] in was conducted in Australia. With one exception [45], all studies were published in English. The mean age of patients varied between 45 and 75.8 years, and the proportion of men ranged from 44.8 to 70.4%. Twelve studies [25, 27, 30-31, 34, 39-45] included only patients with severe sepsis or septic shock. The most frequent source of sepsis was pulmonary infection. Three studies [26, 35, 37] were performed in EDs, one [38a] was performed in a hospital ward, and the remaining studies were performed in an ICU. With respect to admission category characteristics, three studies [25, 28, 33] involved only medical patients, two [34, 39] involved only surgical patients, and the remaining studies involved both medical and surgical patients. Fifteen studies [24-38] evaluated the effect of single PCT concentrations on all-cause mortality in sepsis patients. Among these studies, three [24, 34-35] measured PCT level on the fifth or sixth day after admission and twelve collected blood samples within 24 h of sepsis diagnosis. Eight studies [39-46] evaluated PCT clearance. Follow-up periods differed across studies, including 28 days [25, 29-33], 30 days [26], ICU stays [24, 28] and in-hospital stays [27].

**3.2 Study quality and publication bias**

All studies included a representative sample of patients who underwent PCT testing in practice and clearly described the diagnostic criteria for sepsis. Nine studies [25-28, 33, 36, 39-40, 44] included consecutive patients. Three studies [35, 42, 46] mentioned the blinding of professionals who influenced patient prognosis to PCT level. Four studies [24, 29, 33, 35] excluded patients with comorbidities potentially linked to PCT levels, such as end-organ damage and autoimmune diseases. The Deek’s funnel plot of the included studies, shown in **Figs. 2A** and **2B**, suggested the presence of publication bias.

**3.3 Data synthesis and meta-analysis**

**3.3.1 Analysis of the association of PCT concentration with mortality**

Sixteen studies [24-38] with 3126 patients were included in this group. All studies showed that an elevated PCT level was associated with a higher risk of death, with risk ratios (RR) ranging from 1.38 to 24.62. Because of the substantial heterogeneity between studies (*I*^2^=63.5%), a random-effects model was used to pool RR estimates. The pooled RR was 2.60 (95% CI, 2.05-3.30) (**Fig. 3A**).

No statistically significant difference was observed when exploring for threshold effect (Spearman correlation coefficient=0.062; *P*=0.820). The pooled SEN and SPE were 0.76 (95% CI, 0.67–0.82) and 0.64 (95% CI, 0.52–0.74), respectively (**Fig. 4A**). The PLR and NLR were 2.1 (95% CI, 1.6-2.8) and 0.38 (95% CI, 0.29-0.51), respectively. The DOR was 6 (95% CI, 3-9). The overall area under the SROC curve was 0.77 (95% CI, 0.73–0.80) (**Fig. 5A**).

A univariate meta-regression analysis was performed to explore the sources of potential heterogeneity between studies. The year of publication, the sample size, the prevalence of mortality, the clinical setting, the assay manufacturer, the PCT testing time, the sampling method (i.e., whether studies recruited patients consecutively), and comorbidities (i.e., whether studies excluded patients with comorbidities potentially linked to PCT levels) were included in the analysis. The results indicated that only PCT testing time was statistically significant for heterogeneity (*P*=0.020). The initial PCT level was of limited prognostic value in patients with sepsis. Subgroup analyses are shown in **Table 3**.

**3.3.2 Analysis of the effect of PCT non-clearance on mortality**

Nine studies [39-46] with 868 patients were included in this group. Because the heterogeneity between studies was acceptable (*I*^2^=37.9%), a fixed-effects model was used to pool RR estimates. The pooled RR for mortality was 3.05 (95% CI, 2.35-3.95) (**Fig. 3B**).

No statistically significant differences were observed when exploring the threshold effect (Spearman correlation coefficient=0.133; *P*=0.732). The pooled SEN and SPE were 0.72 (95% CI, 0.58–0.82) and 0.77 (95% CI, 0.55–0.90), respectively (**Fig. 4B**). The PLR and NLR were 3.1 (95% CI, 1.5-6.3) and 0.37 (95% CI, 0.25-0.55), respectively. The DOR was 8 (95% CI, 3-22). The overall area under the SROC curve was 0.79 (95% CI, 0.75–0.83) (**Fig. 5B**).

**4. Discussion**

In this meta-analysis, we first determined that both single PCT concentrations and PCT non-clearance were strongly associated with all-cause mortality in septic patients. Our evidence may confer additional information for the clinical use of PCT apart from diagnosing infection [47] and helping guide therapeutic decision-making [48].

We further identified that PCT non-clearance could predict sepsis mortality. The pooled RR for mortality was 3.05 (95% CI, 2.35-3.95). The overall area under the SROC curve was 0.79 (95% CI, 0.75–0.83). We evaluated the prognostic performance of PCT, and the results showed that the diagnostic performance of both a single PCT concentration and PCT clearance is moderate for predicting sepsis mortality.

The implementation of the appropriate therapeutic interventions appeared to be more significant when initiated rapidly at the time of the patient’s arrival. Delayed resuscitation has been found to be significantly associated with a risk of death [49-50]. The prognostic value of biomarkers have been widely investigated in other meta-analyses [51, 52]. However, those meta-analyses were not immune to unexplained heterogeneity and had a limited number of patients. In our research, PCT non-clearance has emerged as an ideal index to predict prognosis in sepsis. The overall area under the SROC curve was 0.79 (95% CI, 0.75–0.83), which was greater than the results of published meta-analyses of troponins [51] and lactate clearance [52]. In addition, the heterogeneity between studies was acceptable (*I*^2^=37.9%), which showed our results were reliable. The initial PCT level was of limited prognostic value in patients with sepsis. The pooled SEN and SPE were 0.72 (95% CI, 0.63–0.79) and 0.62 (95% CI, 0.49–0.73), respectively. The overall area under the SROC curve was only 0.73 (95% CI, 0.69–0.77).

Our study has several limitations. First, we failed to assess the diagnostic accuracy of PCT for predicting death in ED patients because of the limited number of available studies. Thus, our results probably cannot be applied to ED patients. Second, also owing to the limited number of available studies, we could not perform subgroup analyses based on different admission categories and sites of infection. Third, we could not determine the optimal cut-off value for a single PCT level because we failed to obtain the raw data to map out the ROC curve. Further, we could not conclude the optimal definition of PCT non-clearance required for accurate risk assessment.

Sepsis is a complex pathophysiological process rather than a specific syndrome. Thus far, no ideal biomarker has demonstrated sufficient SEN and SPE to provide clinical utility for predicting sepsis mortality [53]. Clinicians need to provide a more comprehensive evaluation of individual patient conditions. Future studies should highlight the combination of procalcitonin with other clinical indexes as part of an overall assessment of sepsis prognosis rather than adopting a biomarker-based approach to the prediction of sepsis mortality. The combination of PCT and other clinical indexes may provide valuable information to assist clinicians in identifying patients at high risk of dying from sepsis. Several studies [8, 22] showed that PCT concentrations were related to APACHEII and SOFA scores. Of the studies included in this meta-analysis, Suberviola [20] demonstrated an improved prognostic value when PCT was combined with the APACHEII score. Further studies should be performed to help determine the optimal cut-off point and definition for PCT non-clearance required for accurate risk assessment.

**5. Conclusions**

We found that elevated PCT levels and PCT non-clearance were associated with a higher risk of death in patients with sepsis. However, PCT may not be useful as a single index for assessing prognosis because of its moderate diagnostic accuracy, though it may be useful in combination with patients’ overall conditions and other clinical indexes. Further studies are needed to define the optimal cut-off point and a definition of PCT non-clearance required for accurate risk assessment.

**Abbreviations**

PCT=procalcitonin; APACHEII=Acute Physiology and Chronic Health Evaluation; SOFA=Sequential Organ Failure Assessment; ICU=intensive care unit; ED=emergency department; HW=hospital ward; PR=prospective recruitment; CR=consecutive recruitment; RR=retrospective recruitment; RCT=random control trial; MPR=multiple-center prospective recruitment; MRCT=multiple-center random control trial; TP=true positive; FP=false positive; TN=true negative; FN=false negative; SEN=sensitivity; SPE=specificity; PLR=positive likelihood ratio; NLR=negative likelihood ratio; AUC=area under the curve; CI=confidence interval.

**Competing interests**

The authors declare that they have no competing interests.

**Acknowledgments**

We sincerely thank all authors who provided published data for our meta-analysis.

**References**

1. Czura CJ, **“Merinoff Symposium 2010: sepsis”–speaking with one voice**. *Mol Med* 2011, **17**:2–3.

2. Dombrovskiy VY, Martin AA, Sunderram J, Paz HL, **Rapid increase inhospitalization and mortality rates for severe sepsis in the United States: a trend analysis from 1993 to 2003**. *Crit Care Med* 2007, **35**:1244-1250.

3. [Iskander KN](http://www.ncbi.nlm.nih.gov.ezp.lib.unimelb.edu.au/pubmed?term=Iskander KN%5BAuthor%5D&cauthor=true&cauthor_uid=23899564), [Osuchowski MF](http://www.ncbi.nlm.nih.gov.ezp.lib.unimelb.edu.au/pubmed?term=Osuchowski MF%5BAuthor%5D&cauthor=true&cauthor_uid=23899564), [Stearns-Kurosawa DJ](http://www.ncbi.nlm.nih.gov.ezp.lib.unimelb.edu.au/pubmed?term=Stearns-Kurosawa DJ%5BAuthor%5D&cauthor=true&cauthor_uid=23899564), **Sepsis: Multiple Abnormalities, Heterogeneous Responses, and Evolving Understanding**. *Physiol Rev* 2013, **93**:1247-1288.

4. Kiguchi T, **Maximal chemiluminescent intensity in response to lipopolysaccharide assessed by endotoxin activity assay on admission day predicts mortality in patients with sepsis**. *Crit Care Med* 2013, **43**:1443-9.

5. Katharina Gründler, **Platelet mitochondrial membrane depolarization reflects disease severity in patients with sepsis and correlates with clinical outcome**. *Crit Care* 2014, **18**:R31.

6. Behnes M, **Diagnostic and prognostic utility of soluble CD 14 subtype (presepsin) for severe sepsis and septic shock during the first week of intensive care treatment**. *Crit Care* 2014, **14**:507.

7. Akpinar S, **Performance evaluation of MR-proadrenomedullin and other scoring systems in severe sepsis with pneumonia**. *J Thorac Dis* 2014, **6**:921-9.

8. Vassiliou AG, **Elevated biomarkers of endothelial dysfunction/activation at ICU admission are associated with sepsis development**. *Cytokine* 2014, **69**:240-7.

9. HYPERLINK "http://www.ncbi.nlm.nih.gov/pubmed/?term=Kr%C3%BCger S[Author]&cauthor=true&cauthor_uid=17959641" µKrüger S§, **Procalcitonin predicts patients at low risk of death from community-acquired pneumonia across all CRB-65 classes**. HYPERLINK "http://www.ncbi.nlm.nih.gov/pubmed/?term=Procalcitonin+predicts+patients+at+low+risk+of+death+from+community-acquired+pneumonia+across+all+CRB-65+classes" \o "The European respiratory journal." µEur Respir J§ 2008, **31**(2):349-55

10. [Huang DT](http://www.ncbi.nlm.nih.gov/pubmed/?term=Huang DT[Author]&cauthor=true&cauthor_uid=18342993), **Risk Prediction With Procalcitonin and Clinical Rules in Community-Acquired Pneumonia.** [*Ann Emerg Med*](http://www.ncbi.nlm.nih.gov/pubmed/18342993) 2008, **52**(1):48-58.e2

11. HYPERLINK "http://www.ncbi.nlm.nih.gov/pubmed/?term=Hillas G[Author]&cauthor=true&cauthor_uid=19717486" µHillas G§, **C-reactive protein and procalcitonin as predictors of survival and septic shock in ventilator-associated pneumonia**. HYPERLINK "http://www.ncbi.nlm.nih.gov/pubmed/?term=C-reactive+protein+and+procalcitonin+as+predictors+of+survival+and+septic+shock+in+ventilator-associated+pneumonia" \o "The European respiratory journal." µEur Respir J§ 2010, **35**(4):805-11

12. GarnachoMontero J, **Prognostic and diagnostic value of eosinopenia, C-reactive protein, procalcitonin, and circulating cell-free DNA in critically Ill patients admitted with suspicion of sepsis**. *Crit Care* 2014, **18**:R116.

13. Ulla M, **Diagnostic and prognostic value of presepsin in the management of sepsis in the emergency department: a multicenter prospective study**. *Crit Care* 2013, **17**:R168.

14. Levy MM. **2001 SCCM/ESICM/ACCP/ATS/SIS International Sepsis Definitions Conference**. *Intensive Care Med* 2003, **29**:530-8.

15. **American College of Chest Physicians/Society of Critical Care Medicine Consensus Conference: definitions for sepsis and organ failure and guidelines for the use of innovative therapies in sepsis.** *Crit Care* 1992, **20**:864–874.

16. Whiting P, Rutjes AW, Reitsma JB, Bossuyt PM, Kleijnen J. **The development of QUADAS: a tool for the quality assessment of studies of diagnostic accuracy included in systematic reviews**. *J. BMC Med. Res.Methodol.* 2003, **3**: 25.

17. Higgins JPT, **Measuring inconsistency in meta-analyses**. *British Medical Journal* 2003, **327(7414)**: 557.

18. DerSimonian R, **Meta-analysis in clinical trials**. *Control Clin Trials* 1986, **7**:177–188

19. Higgins JP, **Quantifying heterogeneity in a meta-analysis**. *Stat Med* 2002, **21**:1539–1558

20. Cucherat M, **EasyMA: a program for the meta-analysis of clinical trials**. *Comput Methods Programs Biomed* 1997, **53**:187–190

21. Whiting P, **The development of QUADAS: a tool for the quality assessment of studies of diagnostic accuracy included in systematic reviews**. *J. BMC Med. Res.Methodol* 2003, **3**: 25.

22. Reitsma JB, **Bivariate analysis of sensitivity and specificity produces informative summary measures in diagnostic reviews**. *J Clin Epidemiol* 2005, **58**:982-990.

23. Moses LE, Shapiro D, **Combining independent studies of a diagnostic test into a summary ROC curve: data-analytic approaches and some additional considerations**. *Stat Med* 1993, **12**:1293-1316.

24. Adamik B, **Effect of sepsis and cardiac surgery with cardiopulmonary bypass on plasma level of nitric oxide metabolites, neopterin, and procalcitonin: correlation with mortality and postoperative complications**. *Intensive Care Med* 2000, **26**:1259-67.

25. Meng FS, **Serum procalcitonin at the time of admission to the ICU as a predictor of short-term mortality**. *Clin Biochem* 2009, **42**:1025-31.

26. Yin Q, **The role of soluble thrombomodulin in the risk stratification and prognosis evaluation of septic patients in the emergency department**. *Thromb Res* 2013, **132**:471-6.

27. Suberviola B, **Hospital mortality prognostication in sepsis using the new biomarkers suPAR and proADM in a single determination on ICU admission**. *Intensive Care Med* 2013, **39**:1945-52.

28. Clec'h C, **Differential diagnostic value of procalcitonin in surgical and medical patients with septic shock** *Crit Care Med* 2006, **34**:102-7.

29. Li Z, **Serum soluble triggering receptor expressed on myeloid cells-1 and procalcitonin can reflect sepsis severity and predict prognosis: a prospective cohort study** *Mediators Inflamm*, 2014.

30. Masson S, **Presepsin (soluble CD14 subtype) and procalcitonin levels for mortality prediction in sepsis: data from the Albumin Italian Outcome Sepsis trial**. **Crit Care** 2014,**18**: R6.

31. Yaroustovsky M, **Prognostic value of endotoxin activity assay in patients with severe sepsis after cardiac surgery**. *J Inflamm (Lond)* 2013, **10**:8.

32. Feng L, **Clinical significance of soluble hemoglobin scavenger receptor CD163 (sCD163) in sepsis, a prospective study**. *PLoS One* 2012, **7**:e38400.

33. Jain S, **Procalcitonin as a prognostic marker for sepsis: a prospective observational study**. *BMC Res Notes* 2014, **7**: 458-458.

34. Dahaba, A. A, **Procalcitonin for early prediction of survival outcome in postoperative critically ill patients with severe sepsis.** *British Journal of Anaesthesia* 2006, **97**(4): 503-508.

35. Magrini, L, **Procalcitonin variations after Emergency Department admission are highly predictive of hospital mortality in patients with acute infectious diseases.** *European Review for Medical and Pharmacological Sciences* 2013, **17**: 133-142.

36. Savva, A, **Soluble urokinase plasminogen activator receptor (suPAR) for assessment of disease severity in ventilator-associated pneumonia and sepsis**. *Journal of Infection* 2011, **63**(5): 344-350.

37. Kenzaka, T, **Use of a semiquantitative procalcitonin kit for evaluating severity and predicting mortality in patients with sepsis**. *International journal of general medicine* 2012, **5**: 483-488.

38. Giamarellos-Bourboulis, **Procalcitonin as an early indicator of outcome in sepsis: a prospective observational study**. *Journal of Hospital Infection* 2011, **77**(1): 58-63.

39. Tschaikowsky, K, **Predictive value of procalcitonin, interleukin-6, and C-reactive protein for survival in postoperative patients with severe sepsis.** *J Crit Care* 2011, **26**(1): 54-64.

40. Schuetz, P, **Procalcitonin decrease over 72 hours in US critical care units predicts fatal outcome in sepsis patients**. *Crit Care* 2013, **17**(3): R115.

41. Mat Nor, M. B. and A. Md Ralib, **Procalcitonin clearance for early prediction of survival in critically ill patients with severe sepsis.** *Crit Care Res Pract* **2014**: 819034.

42. Ruiz-Rodriguez, J. C., **Usefulness of procalcitonin clearance as a prognostic biomarker in septic shock. A prospective pilot study**. *Medicina Intensiva* 2014, **36**(7): 475-480.

43. Suberviola, B., **Prognostic value of procalcitonin, C-reactive protein and leukocytes in septic shock.** *Medicina Intensiva* 2012, **36**(3): 177-184.

44. Karlsson, S., et al. (2010) Predictive value of procalcitonin decrease in patients with severe sepsis: A prospective observational study. Crit Care **14**, DOI: 10.1186/cc9327

45. Garcia de Guadiana-Romualdo, L. M., et al. (2014). "Prognostic value of lipopolysaccharide binding protein and procalcitonin in patients with severe sepsis and septic shock admitted to intensive care." Med Intensiva.

46. Guan, J., et al. (2011). "Dynamic change of procalcitonin, rather than concentration itself, is predictive of survival in septic shock patients when beyond 10 ng/mL." Shock 36(6): 570-574.

# 47. HYPERLINK "http://www.ncbi.nlm.nih.gov/pubmed/?term=Wacker C[Author]&cauthor=true&cauthor_uid=23375419" µWacker C§1,  HYPERLINK "http://www.ncbi.nlm.nih.gov/pubmed/?term=Prkno A[Author]&cauthor=true&cauthor_uid=23375419" µPrkno A§,  HYPERLINK "http://www.ncbi.nlm.nih.gov/pubmed/?term=Brunkhorst FM[Author]&cauthor=true&cauthor_uid=23375419" µBrunkhorst FM§. Procalcitonin as a diagnostic marker for sepsis: a systematic review and meta-analysis. HYPERLINK "http://www.ncbi.nlm.nih.gov/pubmed/23375419" \o "The Lancet. Infectious diseases." µLancet Infect Dis.§ 2013, 13(5):426-35.

48. Jensen JU, Hein L, Lundgren B, Bestle MH, Mohr TT, Andersen MH, Thornberg KJ, Loken J, Steensen M, Fox Z, Tousi H, Soe-Jensen P, Lauritsen AO, Strange D, Petersen PL, Reiter N, Hestad S, Thormar K, Fjeldborg P, Larsen KM, Drenck NE, Ostergaard C, Kjaer J, Grarup J, Lundgren JD, Procalcitonin And Survival Study (PASS) Group: Procalcitonin-guided interventions against infections to increase early appropriate antibiotics and improve survival in the intensive care unit: a randomized trial. *Crit Care Med* 2011, 39:2048-2058.

49. Ferrer R, Improvement in process of care and outcome after a multicenter severe sepsis educational p**rogram in Spain**. *JAMA* 2008, **299**:2294–2303.

50. [Kiers HD](http://www.ncbi.nlm.nih.gov.ezp.lib.unimelb.edu.au/pubmed?term=Kiers HD%5BAuthor%5D&cauthor=true&cauthor_uid=20646899), **Effect of early achievement of physiologic resuscitation goals in septic patients admitted from the ward on the kidneys**. *J Crit Care* 2010, **24**:563-9.

51. HYPERLINK "http://www.ncbi.nlm.nih.gov/pubmed/?term=Bessi%C3%A8re F[Author]&cauthor=true&cauthor_uid=23595497" µBessière F§,  HYPERLINK "http://www.ncbi.nlm.nih.gov/pubmed/?term=Khenifer S[Author]&cauthor=true&cauthor_uid=23595497" µKhenifer S§,  HYPERLINK "http://www.ncbi.nlm.nih.gov/pubmed/?term=Dubourg J[Author]&cauthor=true&cauthor_uid=23595497" µDubourg J§. **Prognostic value of troponins in sepsis: a meta-analysis**. HYPERLINK "http://www.ncbi.nlm.nih.gov/pubmed/?term=Prognostic+value+of+troponins+in+sepsis:+a+meta-analysis" \o "Intensive care medicine." µIntensive Care Med.§ 2013, **39**(7):1181-9

52. HYPERLINK "http://www.ncbi.nlm.nih.gov/pubmed/?term=Zhang Z[Author]&cauthor=true&cauthor_uid=24797375" µZhang Z§,  HYPERLINK "http://www.ncbi.nlm.nih.gov/pubmed/?term=Xu X[Author]&cauthor=true&cauthor_uid=24797375" µXu X§.Lactate Clearance Is a Useful Biomarker for the Prediction of All-Cause Mortality in Critically Ill Patients: A Systematic Review and Meta-Analysis. HYPERLINK "http://www.ncbi.nlm.nih.gov/pubmed/?term=Lactate+Clearance+Is+a+Useful+Biomarker+for+the+Prediction+of+All-Cause+Mortality+in+Critically+Ill+Patients:+A+Systematic+Review+and+Meta-Analysis" \o "Critical care medicine." µCrit Care Med.§ 2014 Sep;42(9):2118-25

53. Sandquist M, **Biomarkers of sepsis and their potential value in diagnosis, prognosis and treatment**. *Expert Rev Clin Immunol* 2014, **21**:1-8.

**Figure 1**. Flowchart of study selection.

**Figure 2** Deek’s funnel plot asymmetry test for publication bias (A. For single procalcitonin concentration; B. For procalcitonin clearance). Potential publication bias exists (P<0.05).

**Figure 3A** Forest plot of procalcitonin concentration to predict mortality in sepsis. The overall pooled RR was 2.60 (95% CI, 2.05-3.30), suggesting that a higher procalcitonin level was a risk factor of death.

**Figure 3B** Forest plot of procalcitonin clearance to predict mortality in sepsis. The overall pooled RR was 0.33 (95% CI, 0.25-0.43), suggesting that procalcitonin clearance was a factor of survival.

**Figure 4A** Forest plot of the sensitivity and specificity of procalcitonin concentration for predicting mortality in sepsis.

**Figure 4B** Forrest plot of the sensitivity and specificity of procalcitonin clearance for predicting mortality in sepsis.

**Figure 5** Summary receiver operating characteristic graph of the included studies (A. For single procalcitonin concentration; B. For procalcitonin clearance).

**Table 1 Characteristics of studies associating PCT level with mortality**

| Author | Year | Country | Study design | Clinical setting | Follow-up (days) | PCT assay | Testing time | Sample size (n) | Male (%) | Age (years) | Prevalence of mortality (%) | Severity of sepsis | Cut off (ng/ml) | TP | FP | FN | TN | SEN (95% CI) | SPE (95% CI) |
| --- | --- | --- | --- | --- | --- | --- | --- | --- | --- | --- | --- | --- | --- | --- | --- | --- | --- | --- | --- |
| Adamik[24] | 2000 | Poland | PR | ICU | ICU mortality | Lumitest PCT | D5 | 41 | 58.5 | 49.44±15.52 | 61 | Sepsis or severe sepsis | 3 | 25 | 3 | 0 | 13 | 100 | 81 |
| Meng[25] | 2009 | China | PR+CR | MICU | 28-day mortality | PCT-Q | D1 | 86 | 76 | 48.65±15.61 | 37.2 | Severe sepsis | 10 | 24 | 18 | 8 | 36 | 75 | 66.7 |
| Yin[26] | 2013 | China | PR+CR | ED | 30-day mortality | VIDAS | D0 | 680 | 61.2 | 72(60-78) | 33.1 | Sepsis, severe sepsis and septic shock | 0.9 | 139 | 149 | 86 | 306 | 61.8 | 67.3 |
| Suberviola[27] | 2013 | Spain | PR+CR | ICU | In-hospital mortality | KRYPTOR-PCT | D0 | 137 | 65.7 | 62.6±15.9 | 29.9 | Severe sepsis and septic shock | 0.83 | 39 | 82 | 2 | 14 | 95 | 14.4 |
| Clec'h[28] | 2006 | France | PR+CR | MICU | ICU mortality | KRYPTOR-PCT | D1 | 36 | 66.7 | 57±14 | 69.4 | Septic shock | 6 | 19 | 3 | 6 | 8 | 76 | 72.7 |
| Li[29] | 2014 | China | PR | ICU | 28-day mortality | VIDAS | D1 | 102 | 48.4 | 63±21 | 41.2 | - | 10.65 | 36 | 15 | 6 | 45 | 76.2 | 81.7 |
| Masson[30] | 2014 | Italy | MRCT | ICU | 28-day mortality | Cobas® PCT | D1 | 100 | 54 | 71.45±12.34 | 50 | Severe sepsis and septic shock | 14.27 | 28 | 20 | 22 | 30 | 56 | 60 |
| Yaroustovsky[31] | 2013 | Russia | PR | ICU | 28-day mortality | VIDAS | D1 | 81 | 56 | 56(47.5-64.5) | 45.7 | Severe sepsis | 4.76 | 25 | 17 | 12 | 27 | 67 | 62 |
| Feng[32] | 2012 | China | PR | ICU | 28-day mortality | VIDAS | D1 | 102 | 52.2 | 59±19 | 43 | Sepsis and severe sepsis | 8.5 | 30 | 16 | 14 | 42 | 67.4 | 73.2 |
| Jain[33] | 2014 | India | PR+CR | MICU | 28-day mortality | - | D1 | 54 | 55 | 50.68±18.67 | 50.9 | Sepsis, severe sepsis and septic shock | 7 | 13 | 9 | 10 | 22 | 56.6 | 70.9 |
| Dahaba[34] | 2006 | Australia | PR | SICU | 28-day mortality | Lumitest PCT | D6 | 69 | 47.8 | 58(36-77) | 26.1 | Severe sepsis | 3.2 | 15 | 6 | 3 | 45 | 85 | 89 |
| Magrini[35] | 2013 | Italy | PR | ED | In-hospital mortality | VIDAS | D5 | 96 | 41 | 72.6±9.5 | 33.3 | - | - | 28 | 32 | 4 | 32 | 87 | 50 |
| Savva[36] | 2011 | Greece | MPR+CR | ICU | 28-day mortality | KRYPTOR-PCT | D1 | 180 | - | - | 28.3 | Sepsis, severe sepsis and septic shock | 0.92 | 30 | 11 | 21 | 118 | 58.8 | 91.5 |
| Kenzaka[37] | 2012 | Japan | PR | ED | 28-day mortality | PCT-Q | D1 | 206 | 54.9 | 75.8±13.5 | 9.7 | Sepsis, severe sepsis and septic shock | 10 | 11 | 72 | 9 | 114 | 55 | 61.3 |
| Giamarellos-Bourboulis[38a] | 2011 | Greece | MPR | HW | Mortality | KRYPTOR-PCT | D1 | 922 | 51.8 | 65.6±20.6 | 17 | Sepsis, severe sepsis and septic shock | 0.12 | 139 | 558 | 18 | 207 | 88.5 | 27.1 |
| Giamarellos-Bourboulis[38b] | 2011 | Greece | MPR | ICU | Mortality | KRYPTOR-PCT | D1 | 234 | 66.7 | 61.7±17.4 | 35.5 | Sepsis, severe sepsis and septic shock | 0.85 | 53 | 64 | 30 | 87 | 63.9 | 57.6 |

PCT=procalcitonin; ICU=intensive care unit; SICU=surgical intensive care unit; MICU=medical intensive care unit; ED=emergency department; HW=hospital ward; PR=prospective recruitment; CR=consecutive recruitment; RR=retrospective recruitment; RCT=random control trial; MPR=multiple-center prospective recruitment; MRCT= multiple-center random control trial; TP=true positive; FP=false positive; TN=true negative; FN=false negative; SEN=sensitivity; SPE=specificity; CI=confidence interval.

**Table 2 Characteristics of studies associating PCT non-clearance with mortality**

| Author | Year | Country | Study design | Clinical setting | Follow-up (days) | PCT assay | Definition of procalcitonin non-clearance | Sample size (n) | Male (%) | Age (years) | Mortality (%) | Severity of sepsis | TP | FP | FN | TN | SEN (95% CI) | SPE (95% CI) |
| --- | --- | --- | --- | --- | --- | --- | --- | --- | --- | --- | --- | --- | --- | --- | --- | --- | --- | --- |
| Tschaikowsky[39] | 2011 | Germany | PR+CR | SICU | 28-day mortality | KRYPTOR-PCT | PCT↓ < 50% within 7d | 51 | 70.3 | 66.9±10 | 33.3 | Severe sepsis and septic shock | 6 | 1 | 11 | 33 | 35.3 | 97.1 |
| Schuetz[40a] | 2013 | America | RR+CR | ICU | ICU mortality | VIDAS | PCT↓ < 60% within 72 hr | 154 | 44.8 | 65.8±16.2 | 29.2 | Severe sepsis and septic shock | 27 | 36 | 18 | 73 | 60 | 67 |
| Schuetz[40b] | 2013 | America | RR+CR | ICU | ICU mortality | VIDAS | PCT↓ < 60% within 72 hr | 102 | 56.9 | 65.8±17.0 | 17.6 | Severe sepsis and septic shock | 14 | 33 | 4 | 51 | 78 | 61 |
| Mat Nor[41] | 2014 | Malaysia | PR | ICU | In-hospital mortality | KRYPTOR-PCT | PCT↓ < 30% within 48 hr | 67 | 70.1 | 45±16 | 40.3 | Severe sepsis | 20 | 18 | 7 | 22 | 74.1 | 55 |
| Ruiz-Rodriguez[42] | 2012 | Spain | PR | ICU | ICU mortality | Lumitest PCT | PCT↓ < 50% within 48 hr | 27 | 70.4 | 65.7±14 | 66.7 | Septic shock | 8 | 5 | 1 | 13 | 89 | 72 |
| Suberviola[43] | 2012 | Spain | PR | ICU | In-hospital mortality | KRYPTOR-PCT | PCT↓ < 70% within 72 hr | 88 | 63.6 | 64.8 ± 18.7 | 23.9 | Septic shock | 10 | 4 | 9 | 65 | 52.6 | 94.2 |
| Karlsson[44] | 2010 | Finland | PR+CR | ICU | In-hospital mortality | Cobas® PCT | PCT↓ < 50% within 72 hr | 242 | 68.2 | 59.8±15.4 | 24.2 | Severe sepsis | 55 | 130 | 7 | 50 | 88.7 | 27.8 |
| Garcia de Guadiana-Romualdo[45] | 2014 | Spain | PR | ICU | In-hospital mortality | Cobas® PCT | PCT↓ < 40% within 48 hr | 100 | 53 | 67±56 | 28 | Severe sepsis and septic shock | 18 | 27 | 10 | 45 | 64.3 | 62.5 |
| Guan[46] | 2011 | China | PR | ICU | Mortality | Lumitest PCT | PCT↓ < 25% within 5d | 37 | 59 | 56±33.5 | 32.4 | Sepsis, severe sepsis and septic shock | 12 | 0 | 0 | 25 | 100 | 100 |

PCT=procalcitonin; ICU=intensive care unit; SICU=surgical intensive care unit; ED=emergency department; HW=hospital ward; PR=prospective recruitment; CR=consecutive recruitment; RR=retrospective recruitment; RCT=random control trial; MPR=multiple-center prospective recruitment; MRCT=multiple-center random control trial; TP=true positive; FP=false positive; TN=true negative; FN=false negative; SEN=sensitivity; SPE=specificity; CI=confidence interval.

**Table 3. Subgroup analysis**

| Variables | No. of studies | No. of patients | SEN (95% CI) | SPE (95% CI) | DOR (95% CI) | PLR (95% CI) | NLR (95% CI) | AUC (95% CI) | Test for heterogeneity (I2) | Deek's funnel test (p value) |
| --- | --- | --- | --- | --- | --- | --- | --- | --- | --- | --- |
| Overall | 16 | 3126 | 0.76(0.67-0.82) | 0.64(0.52-0.74) | 6(3-9) | 2.1(1.6-2.8) | 0.38(0.29-0.51) | 0.77(0.73-0.80) | 63.5 | 0.04 |
| Initial PCT concentration | 13 | 2920 | 0.72(0.63-0.79) | 0.62(0.49-0.73) | 4(3-6) | 1.9(1.4-2.4) | 0.46(0.37-0.56) | 0.73(0.69-0.77) | 57.5 | 0.29 |
| ICU patients | 12 | 1222 | 0.76(0.65-0.84) | 0.69(0.55-0.80) | 7(4-13) | 2.4(1.7-3.6) | 0.35(0.24-0.51) | 0.79(0.75-0.82) | 72.1 | 0.12 |
| Severe sepsis/septic shock | 6 | 509 | 0.77(0.62-0.87) | 0.61(0.38-0.80) | 5(2-11) | 1.9(1.2-3.2) | 0.39(0.25-0.61) | 0.76(0.73-0.80) | 67.3 | 0.17 |

PCT=procalcitonin; ICU=intensive care unit; ED=emergency department; SEN=sensitivity; SPE=specificity; DOR=diagnostic odds ratio; PLR=positive likelihood ratio; NLR=negative likelihood ratio; AUC=area under the curve; CI=confidence interval.
