# Supplementary material for: Prognostic Value of Procalcitonin in Adult Patients with Sepsis: A Systematic Review and Meta-Analysis
Source: PLoS One. 2015 Jun 15;10(6):e0129450. doi: 10.1371/journal.pone.0129450 (PMC4468164; doi:10.1371/journal.pone.0129450)
Supplement: S4 File — (DOC) [file pone.0129450.s004.doc]

**Table 1 Characteristics of studies associating PCT level with mortality**

| Author | Year | Study design | Clinical setting | Follow-up (days) | PCT assay | Testing time | Sample size (n) | Prevalence of mortality (%) | Severity of sepsis | Cut off (ng/ml) | SEN (95% CI) | SPE (95% CI) |
| --- | --- | --- | --- | --- | --- | --- | --- | --- | --- | --- | --- | --- |
| Adamik[24] | 2000 | PR | ICU | ICU mortality | Lumitest PCT | D5 | 41 | 61 | Sepsis or severe sepsis | 3 | 100 | 81 |
| Meng[25] | 2009 | PR+CR | MICU | 28-day mortality | PCT-Q | D1 | 86 | 37.2 | Severe sepsis | 10 | 75 | 66.7 |
| Yin[26] | 2013 | PR+CR | ED | 30-day mortality | VIDAS | D0 | 680 | 33.1 | Sepsis, severe sepsis and septic shock | 0.9 | 61.8 | 67.3 |
| Suberviola[27] | 2013 | PR+CR | ICU | In-hospital mortality | KRYPTOR-PCT | D0 | 137 | 29.9 | Severe sepsis and septic shock | 0.83 | 95 | 14.4 |
| Clec'h[28] | 2006 | PR+CR | MICU | ICU mortality | KRYPTOR-PCT | D1 | 36 | 69.4 | Septic shock | 6 | 76 | 72.7 |
| Li[29] | 2014 | PR | ICU | 28-day mortality | VIDAS | D1 | 102 | 41.2 | - | 10.65 | 76.2 | 81.7 |
| Masson[30] | 2014 | MRCT | ICU | 28-day mortality | Cobas PCT | D1 | 100 | 50 | Severe sepsis and septic shock | 14.27 | 56 | 60 |
| Yaroustovsky[31] | 2013 | PR | ICU | 28-day mortality | VIDAS | D1 | 81 | 45.7 | Severe sepsis | 4.76 | 67 | 62 |
| Feng[32] | 2012 | PR | ICU | 28-day mortality | VIDAS | D1 | 102 | 43 | Sepsis and severe sepsis | 8.5 | 67.4 | 73.2 |
| Jain[33] | 2014 | PR+CR | MICU | 28-day mortality | - | D1 | 54 | 50.9 | Sepsis, severe sepsis and septic shock | 7 | 56.6 | 70.9 |
| Dahaba[34] | 2006 | PR | SICU | 28-day mortality | Lumitest PCT | D6 | 69 | 26.1 | Severe sepsis | 3.2 | 85 | 89 |
| Magrini[35] | 2013 | PR | ED | In-hospital mortality | VIDAS | D5 | 96 | 33.3 | - | - | 87 | 50 |
| Savva[36] | 2011 | MPR+CR | ICU | 28-day mortality | KRYPTOR-PCT | D1 | 180 | 28.3 | Sepsis, severe sepsis and septic shock | 0.92 | 58.8 | 91.5 |
| Kenzaka[37] | 2012 | PR | ED | 28-day mortality | PCT-Q | D1 | 206 | 9.7 | Sepsis, severe sepsis and septic shock | 10 | 55 | 61.3 |
| Giamarellos-Bourboulis[38a] | 2011 | MPR | HW | Mortality | KRYPTOR-PCT | D1 | 922 | 17 | Sepsis, severe sepsis and septic shock | 0.12 | 88.5 | 27.1 |
| Giamarellos-Bourboulis[38b] | 2011 | MPR | ICU | Mortality | KRYPTOR-PCT | D1 | 234 | 35.5 | Sepsis, severe sepsis and septic shock | 0.85 | 63.9 | 57.6 |

PCT=procalcitonin; ICU=intensive care unit; SICU=surgical intensive care unit; MICU=medical intensive care unit; ED=emergency department; HW=hospital ward; PR=prospective recruitment; CR=consecutive recruitment; RR=retrospective recruitment; RCT=random control trial; MPR=multiple-center prospective recruitment; MRCT= multiple-center random control trial; SEN=sensitivity; SPE=specificity; CI=confidence interval.

**Table 2 Characteristics of studies associating PCT non-clearance with mortality**

| Author | Year | Study design | Clinical setting | Follow-up (days) | PCT assay | Definition of procalcitonin non-clearance | Sample size (n) | Mortality (%) | Severity of sepsis | SEN (95% CI) | SPE (95% CI) |
| --- | --- | --- | --- | --- | --- | --- | --- | --- | --- | --- | --- |
| Tschaikowsky[39] | 2011 | PR+CR | SICU | 28-day mortality | KRYPTOR-PCT | PCT↓ < 50% within 7d | 51 | 33.3 | Severe sepsis and septic shock | 35.3 | 97.1 |
| Schuetz[40a] | 2013 | RR+CR | ICU | ICU mortality | VIDAS | PCT↓ < 60% within 72 hr | 154 | 29.2 | Severe sepsis and septic shock | 60 | 67 |
| Schuetz[40b] | 2013 | RR+CR | ICU | ICU mortality | VIDAS | PCT↓ < 60% within 72 hr | 102 | 17.6 | Severe sepsis and septic shock | 78 | 61 |
| Mat Nor[41] | 2014 | PR | ICU | In-hospital mortality | KRYPTOR-PCT | PCT↓ < 30% within 48 hr | 67 | 40.3 | Severe sepsis | 74.1 | 55 |
| Ruiz-Rodriguez[42] | 2012 | PR | ICU | ICU mortality | Lumitest PCT | PCT↓ < 50% within 48 hr | 27 | 66.7 | Septic shock | 89 | 72 |
| Suberviola[43] | 2012 | PR | ICU | In-hospital mortality | KRYPTOR-PCT | PCT↓ < 70% within 72 hr | 88 | 23.9 | Septic shock | 52.6 | 94.2 |
| Karlsson[44] | 2010 | PR+CR | ICU | In-hospital mortality | Cobas PCT | PCT↓ < 50% within 72 hr | 242 | 24.2 | Severe sepsis | 88.7 | 27.8 |
| Garcia de Guadiana-Romualdo[45] | 2014 | PR | ICU | In-hospital mortality | Cobas PCT | PCT↓ < 40% within 48 hr | 100 | 28 | Severe sepsis and septic shock | 64.3 | 62.5 |
| Guan[46] | 2011 | PR | ICU | Mortality | Lumitest PCT | PCT↓ < 25% within 5d | 37 | 32.4 | Sepsis, severe sepsis and septic shock | 100 | 100 |

PCT=procalcitonin; ICU=intensive care unit; SICU=surgical intensive care unit; ED=emergency department; HW=hospital ward; PR=prospective recruitment; CR=consecutive recruitment; RR=retrospective recruitment; RCT=random control trial; MPR=multiple-center prospective recruitment; MRCT=multiple-center random control trial; SEN=sensitivity; SPE=specificity; CI=confidence interval.

**Table 3. Subgroup analysis**

| Variables | No. of studies | No. of patients | SEN (95% CI) | SPE (95% CI) | DOR (95% CI) | PLR (95% CI) | NLR (95% CI) | AUC (95% CI) | Test for heterogeneity (I2) | Deek's funnel test (p value) |
| --- | --- | --- | --- | --- | --- | --- | --- | --- | --- | --- |
| Overall | 16 | 3126 | 0.76(0.67-0.82) | 0.64(0.52-0.74) | 6(3-9) | 2.1(1.6-2.8) | 0.38(0.29-0.51) | 0.77(0.73-0.80) | 63.5 | 0.04 |
| Initial PCT concentration | 13 | 2920 | 0.72(0.63-0.79) | 0.62(0.49-0.73) | 4(3-6) | 1.9(1.4-2.4) | 0.46(0.37-0.56) | 0.73(0.69-0.77) | 57.5 | 0.29 |
| ICU patients | 12 | 1222 | 0.76(0.65-0.84) | 0.69(0.55-0.80) | 7(4-13) | 2.4(1.7-3.6) | 0.35(0.24-0.51) | 0.79(0.75-0.82) | 72.1 | 0.12 |
| Severe sepsis/septic shock | 6 | 509 | 0.77(0.62-0.87) | 0.61(0.38-0.80) | 5(2-11) | 1.9(1.2-3.2) | 0.39(0.25-0.61) | 0.76(0.73-0.80) | 67.3 | 0.17 |

PCT=procalcitonin; ICU=intensive care unit; ED=emergency department; SEN=sensitivity; SPE=specificity; DOR=diagnostic odds ratio; PLR=positive likelihood ratio; NLR=negative likelihood ratio; AUC=area under the curve; CI=confidence interval.
