# Supplementary figures and images for: Prognostic Value of Procalcitonin in Adult Patients with Sepsis: A Systematic Review and Meta-Analysis
Source: PLoS One. 2015 Jun 15;10(6):e0129450. doi: 10.1371/journal.pone.0129450 (PMC4468164; doi:10.1371/journal.pone.0129450)

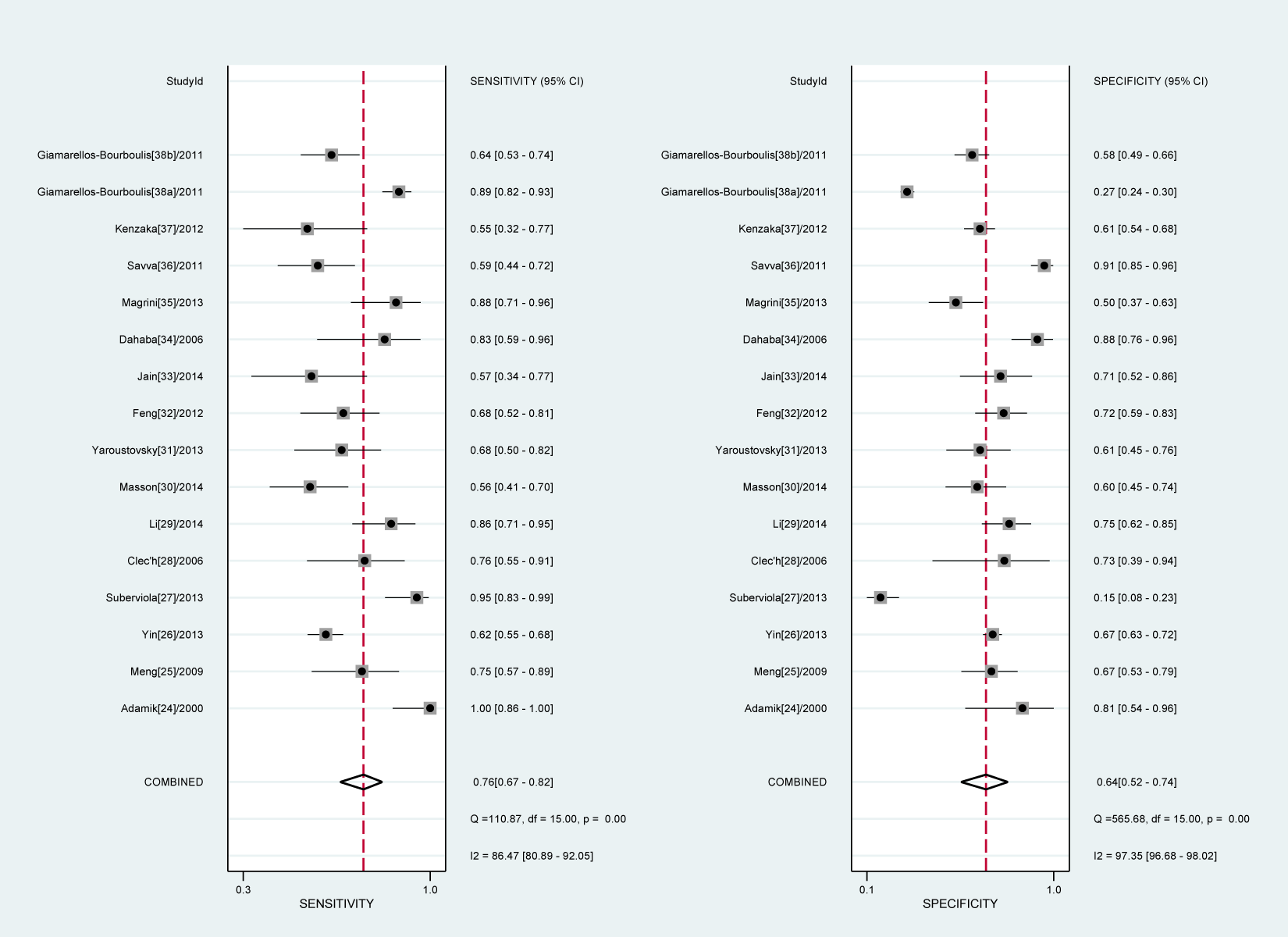

Supplement: S1 Fig — (TIF) [file pone.0129450.s005.tif]

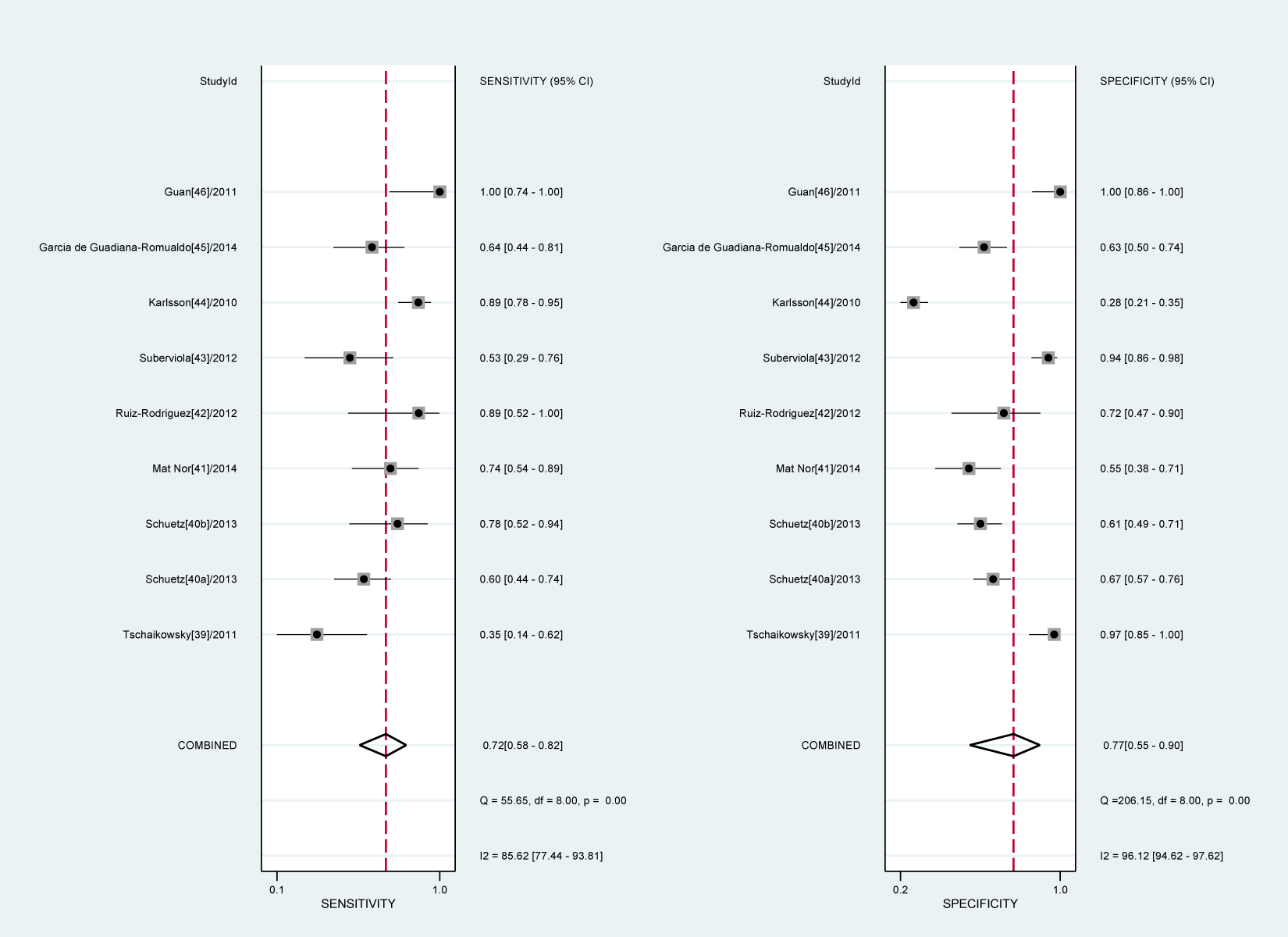

Supplement: S2 Fig — (TIF) [file pone.0129450.s006.tif]
